# Impact of COVID-19 on surgical emergencies: nationwide analysis

**DOI:** 10.1093/bjsopen/zrab039

**Published:** 2021-05-22

**Authors:** A Lazzati, M Raphael Rousseau, S Bartier, Y Dabi, A Challine, B Haddad, N Herta, E Souied, M Ortala, S Epaud, M Masson, N Salaün-Penquer, A Coste, C Jung

**Affiliations:** 1 Department of General and Digestive Surgery, Intercommunal Hospital of Créteil, Créteil, France; 2 INSERM U955, IMRB, Créteil, France; 3 Department of Medical Informatics, Intercommunal Hospital of Créteil, Créteil, France; 4 University Paris-Est Creteil, School of Medicine, Créteil, France; 5 Department of Oto-rhino-laryngology Head and Neck Surgery, Intercommunal Hospital of Créteil, Créteil, France; 6 Department of Oto-rhino-laryngology Head and Neck Surgery, Paris Public Hospitals, Henri Mondor Hospital, France; 7 CNRS, ERL 7240, Créteil, France; 8 Department of Obstetrics and Gynaecology, Intercommunal Hospital of Créteil, Créteil, France; 9 Department of Digestive, Hepatobiliary and Pancreatic Surgery, AP-HP, Université de Paris, Cochin Hospital, France; 10 Department of Ophthalmology, Intercommunal Hospital of Créteil, Créteil, France; 11 Kaduceo SAS, Toulouse, France; 12 Clinical Research Centre, Intercommunal Hospital of Créteil, Créteil, France

## Abstract

**Background:**

The COVID-19 pandemic has had a major impact on healthcare in many countries. This study assessed the effect of a nationwide lockdown in France on admissions for acute surgical conditions and the subsequent impact on postoperative mortality.

**Methods:**

This was an observational analytical study, evaluating data from a national discharge database that collected all discharge reports from any hospital in France. All adult patients admitted through the emergency department and requiring a surgical treatment between 17 March and 11 May 2020, and the equivalent period in 2019 were included. The primary outcome was the change in number of hospital admissions for acute surgical conditions. Mortality was assessed in the matched population, and stratified by region.

**Results:**

During the lockdown period, 57 589 consecutive patients were admitted for acute surgical conditions, representing a decrease of 20.9 per cent compared with the 2019 cohort. Significant differences between regions were observed: the decrease was 15.6, 17.2, and 26.8 per cent for low-, intermediate- and high-prevalence regions respectively. The mortality rate was 1.92 per cent during the lockdown period and 1.81 per cent in 2019. In high-prevalence zones, mortality was significantly increased (odds ratio 1.22, 95 per cent c.i. 1.06 to 1.40).

**Conclusion:**

A marked decrease in hospital admissions for surgical emergencies was observed during the lockdown period, with increased mortality in regions with a higher prevalence of COVID-19 infection. Health authorities should use these findings to preserve quality of care and deliver appropriate messages to the population.

## Teaser

This study assessed the change in acute surgical conditions during the lockdown period on a nationwide scale. There was an overall reduction in emergency operations of 20.9 per cent, with great variability according to prevalence of COVID-19 admissions. Mortality increased in regions with a high prevalence of COVID-19 infection.

## Introduction

The severe acute respiratory syndrome coronavirus 2 (SARS-CoV-2; COVID-19) pandemic has had profound effects on healthcare systems globally. Hospitals, in particular, have been overwhelmed by the massive influx of infected patients. To cope with the burden of disease, hospital workforce was reallocated and elective surgery significantly delayed. Various countries implemented a national lockdown, with major restrictions on all non-essential travel outside the home. In France, an initial lockdown was declared from 17 March to 11 May 2020^1^.

Few studies have reported on the impact on emergency department visits for acute illnesses not related to COVID-19 during the lockdown period, although decreased attendances have been described for myocardial infarction, trauma, and acute gastrointestinal conditions including appendicitis and acute cholecystitis^1–4^.

Although individual centres and specialties rapidly identified the impact of COVID-19 on surgical services, there remains a lack of information on its effect on emergency surgery at a nationwide level during lockdown.

This study investigated how the sudden disruption of usual healthcare during the lockdown period affected acute surgery. The aim was to quantify changes in hospital admissions for emergency surgical conditions according to the regional prevalence of COVID-19, comparing the lockdown period with the same time interval in 2019. Potential changes in mortality were investigated.

## Methods

###  

This was an observational, analytical study of the impact of a national lockdown during the SARS-CoV-2 pandemic on the rate of surgical emergencies. Data were extracted from a national discharge database, the Programme De Médicalisation des Systèmes d’Information (PMSI), which collects all discharge reports from all hospitals in France, irrespective of facility ownership or academic affiliation. Discharge reports are mandatory and represent the basis for hospital funding. The database is comprehensive for all reimbursed surgical interventions in the country.

Data collected included patient demographics (age, sex, postal code, admission and discharge dates) along with primary and associated diagnoses based on ICD-10.

### Participants

All adult patients aged at least 18  years admitted during the period of lockdown between 17 March and 11 May 2020 and the equivalent period in 2019 (19 March and 13 May) were considered. Patients were identified in the database through the diagnosis-related group classification, used to identify any hospital stay in which a surgical event occurred. Only emergency admissions were considered, defined as any admission passing through the emergency department. In the case of multiple admissions for the same patient, all hospital stays were included.

### Exposures and confounders

The exposure variable was the year of admission, 2019 *versus* 2020, the year 2019 being the reference group. Potential confounders in readmission destination were assessed at several levels. Baseline patient characteristics included age, sex, BMI, and co-morbidities, according to the Charlson Co-morbidity Index (using Bannay weighting)^5^.

Regional differences were based on the reported ratios of hospital admissions for COVID-19 infection per 100 000 inhabitants. Three regional groups were established based on the numbers of admissions: 30 or more per 100 000 in high-prevalence regions, 15–29 per 100 000 in intermediate-prevalence regions, and fewer than 15 per 100 000 in low-prevalence regions^2^.

In the ICD-10 catalogue, diagnosis codes have a hierarchical classification in four levels^6^ based on 22 chapters, each using a letter code. Each chapter is divided into blocks of homogenous three-character categories (for instance, codes K35–K38 represent diseases of appendix). In this study, these two first levels of classification are referred to as chapters and blocks. Within each block, ICD-10 codes are classified into three-character categories (K35 represents acute appendicitis) and four-character subcategories (K35.2 represents acute appendicitis with generalized peritonitis), defining disease characteristics in increased detail. In this study, the last four-character level is referred to as a subcategory.

In the present study, 90 per cent of the most frequent diagnoses using the four-character subcategories of ICD-10 codes were selected, reducing the number of diagnoses from over 10 000 to approximately 500. Complete attrition is reported in *Fig.S1*.

### Outcomes

The main outcome of this study was the rate of admission for adult surgical emergencies during the lockdown period in France compared with the same interval in 2019. A secondary outcome was in-hospital mortality after admission. Mortality was assessed irrespective of the time between the day of admission and death. The impact of active SARS-CoV-2 infection on mortality was assessed in a subgroup analysis.

### Data access and linkage

In the PMSI database, each patient is assigned a unique identifier, which remains unchanged over time, making linkage between hospital stays in different hospitals possible. Because the identifier is anonymous, patient consent was not required. Access to the database was submitted for authorization by the National Commission on Informatics and Liberty (authorization number 01947391).

### Statistical analysis

The balance among patient co-variables was assessed using standardized mean differences (SMDs); a difference of 10 per cent or less was considered a well balanced result^7^. The paired-samples Wilcoxon signed-rank test was used to examine the difference in median number of emergencies between lockdown and control periods.

Potential confounders among measured co-variables were assessed by propensity score analysis. The probability of each patient being admitted during the lockdown was calculated by logistic regression incorporating all patient variables. Matching between the lockdown and control groups was performed using the nearest neighbour for propensity score and the exact method for the diagnosis code (using the 3-character category), sex, and age group. In the matched cohort, the balance between co-variables was also assessed using the SMD. Mortality odds ratios (ORs) for each surgical disease were estimated by means of a logistic univariable regression model.

A similar method was used to calculate the OR for mortality associated with COVID-19. Patients with COVID-19 from the lockdown period were matched with those admitted during the same interval using the propensity score, as described above. An adjusted OR for mortality with confidence interval was calculated using the logistic regression model. All statistical analyses were done with R software (R Foundation for Statistical Computing, Vienna, Austria).

## Results

###  

**Fig. 1:**
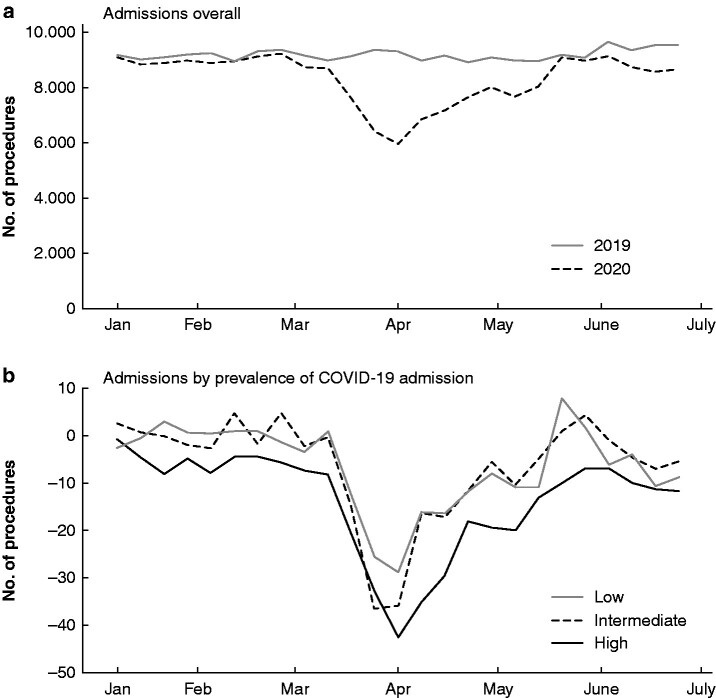
Acute surgical admissions **a** Overall and **b** according to regional prevalence of COVID-19 admission. The shaded area represents the period of lockdown in 2020.

During the lockdown, 57 589 emergency surgical admissions occurred in France, representing a decrease of 20.9 per cent compared with the same period in 2019 (72 819 admission). The nadir of admissions was observed during week 12 (–36.1 per cent), followed by gradual increases, until the first week after the end of lockdown (week 20), when the difference between 2019 and 2020 was negligible (*Fig. 1a*).

The decrease in emergency surgical admissions differed between regions, reflecting the overall prevalence of admissions for COVID-19 infection. This amounted to 15.6 and 17.2 per cent decreases for low- and intermediate-prevalence regions respectively, with a 26.8 per cent decrease for high-prevalence regions where the nadir in week 13 was 42.3 per cent (*Fig. 1b*).

The characteristics of patients admitted during the lockdown were similar to those of patients admitted during the same interval in 2019, with a mean(?) SMD of 0.015(0.013); no co-variable had a SMD larger than 0.100 (*Table 1*).

**Table 1:** Baseline characteristics

	**Control group (2019)** **(*n* = 72 819)**	**Lockdown group (2020)** **(*n* = 57 589)**	SMD
Age (years)*	56.49(23.08)	57.34(23.01)	0.037
< 30	13 104 (18.0)	9461 (16.4)	
30–39	10 611 (14.6)	8744 (15.2)	
40–49	7203 (9.9)	5658 (9.8)	
50–59	8127 (11.2)	6318 (11.0)	
60–75	14 294 (19.6)	11 178 (19.4)	
> 75	19 501 (26.8)	16 230 (28.2)	
**Women**	40 452 (55.5)	32 466 (56.4)	0.017
Charlson Co-morbidity Index score*	0.42 (0.87)	0.44 (0.89)	0.026
0	54 382 (74.7)	42 275 (73.4)	
1–2	15 578 (21.4)	12 856 (22.3)	
>3	2880 (4.0)	2458 (4.3)	
**Myocardial infarction**	875 (1.2)	676 (1.2)	0.003
**Congestive heart failure**	3698 (5.1)	3140 (5.5)	0.017
**Peripheral vascular disease**	2046 (2.8)	1738 (3.0)	0.012
**Cerebrovascular disease**	1700 (2.3)	1352 (2.3)	0.001
**Dementia**	3027 (4.2)	2393 (4.2)	<0.001
**Chronic pulmonary disease**	2413 (3.3)	2228 (3.9)	0.030
**Rheumatic disease**	350 (0.5)	300 (0.5)	0.006
**Peptic ulcer disease**	427 (0.6)	317 (0.6)	0.005
**Mild liver disease**	684 (0.9)	608 (1.1)	0.012
**Diabetes without chronic complication**	5172 (7.1)	4276 (7.4)	0.013
**Diabetes with chronic complication**	1399 (1.9)	1009 (1.8)	0.013
**Hemiplegia or paraplegia**	1281 (1.8)	1014 (1.8)	<0.001
**Renal disease**	2518 (3.5)	2119 (3.7)	0.012
**Any malignancy, including lymphoma and leukaemia, except malignant neoplasm of skin**	3330 (4.6)	2933 (5.1)	0.024
**Moderate or severe liver disease**	156 (0.2)	154 (0.3)	0.011
**Metastatic solid tumour**	1321 (1.8)	1119 (1.9)	0.01
**AIDS/HIV**	81 (0.1)	54 (0.1)	0.005
**Obesity**	3588 (4.9)	3026 (5.3)	0.015

Values in parentheses are percentages unless indicated otherwise; *values are mean(s.d.). SMD, standardized mean difference; AIDS/HIV, acquired immune deficiency syndrome/human imuunodeficiency virus.

Trends in admission by chapter and category are reported in *Table 2*. The decrease in number of emergency admissions affected all chapters, except other reasons for admission, where numbers were relatively small. Admissions related to the injury and digestive system chapters were the most prevalent, and decreases of 27 and 19 per cent respectively were noted (*P* < 0.001). Chapters that had the greatest decrease were eye and adnexa (–40.5 per cent; *P* = 0.002) and respiratory system (–40.7 per cent; *P* < 0.001), whereas the least affected were neoplasms and pregnancy (8.5 and 7.5 per cent decrease respectively; *P* = 0.032 and 0.014).

**Table 2:** Surgical emergencies classified by chapter and category

Chapter	Block code	Block label	Control group (2019)	**Lockdown group (2020)**	**Difference** **(%)**	*P*
Infectious diseases	A30–A49	Other bacterial diseases	111 (0.15)	72 (0.13)	–35.14	0.014
	Total		111 (0.15)	72 (0.13)	–35.14	0.016
Neoplasms	C15–C26	Malignant neoplasms, digestive organs	671 (0.92)	641 (1.11)	–4.47	0.433
	C50–C58	Malignant neoplasms, breast and female genital organs	65 (0.09)	42 (0.07)	–35.38	0.040
	C60–C63	Malignant neoplasms of male genital organs	38 (0.05)	42 (0.07)	10.53	0.687
	C64–C68	Malignant neoplasms, urinary organs	276 (0.38)	263 (0.46)	–4.71	0.581
	C69–C72	Malignant neoplasms, eye, brain, and central nervous system	47 (0.06)	34 (0.06)	–27.66	0.206
	C76–C80	Malignant neoplasms, secondary and ill defined	217 (0.3)	186 (0.32)	–14.29	0.087
	C81–C96	Malignant neoplasms, stated or presumed to be primary, of lymphoid, haematopoietic, and related tissue	47 (0.06)	42 (0.07)	–10.64	0.521
	D10–D36	Benign neoplasms	99 (0.14)	85 (0.15)	–14.14	0.395
	Total		1460 (2)	1335 (2.32)	–8.56	0.032
Nervous system	G00–G09	Inflammatory diseases of the central nervous system	25 (0.03)	18 (0.03)	–28	0.404
	G50–G59	Nerve, nerve root, and plexus disorders	76 (0.1)	49 (0.09)	–35.53	0.011
	G90–G99	Other disorders of the nervous system	25 (0.03)	9 (0.02)	–64	0.017
	Total		126 (0.17)	76 (0.13)	–39.68	0.003
Eye and adnexa	H15–H19	Disorders of sclera and cornea	134 (0.18)	57 (0.1)	–57.46	0.083
	H25–H28	Disorders of lens	26 (0.04)	0 (0)	–100	<0.001
	H30–H36	Disorders of choroid and retina	165 (0.23)	147 (0.26)	–10.91	0.536
	H43–H45	Disorders of vitreous body and globe	70 (0.1)	31 (0.05)	–55.71	0.031
	Total		395 (0.54)	235 (0.41)	–40.51	0.002
Circulatory system	I20–I25	Ischaemic heart diseases	285 (0.39)	187 (0.32)	–34.39	<0.001
	I30–I52	Other forms of heart disease	1841 (2.53)	1480 (2.57)	–19.61	<0.001
	I60–I69	Cerebrovascular diseases	322 (0.44)	264 (0.46)	–18.01	0.031
	I70–I79	Diseases of arteries, arterioles, and capillaries	1011 (1.39)	897 (1.56)	–11.28	0.051
	Total		3459 (4.75)	2828 (4.91)	–18.24	<0.001
Respiratory system	J30–J39	Other diseases of upper respiratory tract	304 (0.42)	151 (0.26)	–50.33	<0.001
	J85–J86	Suppurative and necrotic conditions of lower respiratory tract	24 (0.03)	17 (0.03)	–29.17	0.26
	J90–J94	Other diseases of pleura	185 (0.25)	136 (0.24)	–26.49	0.005
	Total		513 (0.7)	304 (0.53)	–40.74	<0.001
Digestive system	K00–K14	Diseases of oral cavity, salivary glands, and jaws	278 (0.38)	165 (0.29)	–40.65	0.004
	K20–K31	Diseases of oesophagus, stomach, and duodenum	179 (0.25)	157 (0.27)	–12.29	0.204
	K35–K38	Diseases of appendix	5520 (7.58)	4357 (7.57)	–21.07	<0.001
	K40–K46	Hernia	1614 (2.22)	1209 (2.1)	–25.09	<0.001
	K55–K63	Other diseases of intestines	3528 (4.84)	2789 (4.84)	–20.95	<0.001
	K65–K67	Diseases of peritoneum	494 (0.68)	343 (0.6)	–30.57	<0.001
	K80–K87	Disorders of gallbladder, biliary tract, and pancreas	3292 (4.52)	3107 (5.4)	–5.62	0.089
	K90–K93	Other diseases of the digestive system	75 (0.1)	46 (0.08)	–38.67	0.016
	Total		14 980 (20.57)	12 173 (21.14)	–18.74	<0.001
Skin and subcutaneous tissue	L00–L08	Infections of the skin and subcutaneous tissue	2557 (3.51)	1773 (3.08)	–30.66	<0.001
	L60–L75	Disorders of skin appendages	137 (0.19)	104 (0.18)	–24.09	0.04
	L80–L99	Other disorders of the skin and subcutaneous tissue	123 (0.17)	96 (0.17)	–21.95	0.13
	Total		2817 (3.87)	1973 (3.43)	–29.96	<0.001
Musculoskeletal system and connective tissue	M00–M03	Infectious arthropathies	638 (0.88)	483 (0.84)	–24.29	<0.001
	M15–M19	Arthrosis	37 (0.05)	12 (0.02)	–67.57	0.004
	M20–M25	Other joint disorders	29 (0.04)	6 (0.01)	–79.31	<0.001
	M45–M49	Spondylopathies	46 (0.06)	47 (0.08)	2.17	0.942
	M50–M54	Other dorsopathies	224 (0.31)	226 (0.39)	0.89	0.941
	M65–M68	Disorders of synovium and tendon	600 (0.82)	475 (0.82)	–20.83	0.007
	M70–M79	Other soft tissue disorders	371 (0.51)	348 (0.6)	–6.2	0.469
	M80–M85	Disorders of bone density and structure	126 (0.17)	82 (0.14)	–34.92	<0.001
	M86–M90	Other osteopathies	437 (0.6)	282 (0.49)	–35.47	<0.001
	M95–M99	Other disorders of the musculoskeletal system and connective tissue	558 (0.77)	454 (0.79)	–18.64	0.012
	Total		3066 (4.21)	2415 (4.19)	–21.23	<0.001
Genitourinary system	N00–N08	Glomerular diseases	24 (0.03)	14 (0.02)	–41.67	0.096
	N10–N16	Renal tubulointerstitial diseases	1801 (2.47)	1714 (2.98)	–4.83	0.284
	N17–N19	Renal failure	159 (0.22)	139 (0.24)	–12.58	0.265
	N20–N23	Urolithiasis	2572 (3.53)	2590 (4.5)	0.7	0.860
	N25–N29	Other disorders of kidney and ureter	27 (0.04)	32 (0.06)	18.52	0.442
	N30–N39	Other diseases of urinary system	90 (0.12)	56 (0.1)	–37.78	0.012
	N40–N51	Diseases of male genital organs	642 (0.88)	446 (0.77)	–30.53	<0.001
	N60–N64	Disorders of breast	24 (0.03)	21 (0.04)	–12.5	0.655
	N70–N77	Inflammatory diseases of female pelvic organs	720 (0.99)	539 (0.94)	–25.14	<0.001
	N80–N98	Non-inflammatory disorders of female genital tract	408 (0.56)	293 (0.51)	–28.19	<0.001
	Total		6467 (8.88)	5844 (10.15)	–9.63	<0.001
Pregnancy, childbirth, and the puerperium	O00–O08	Pregnancy with abortive outcome	2161 (2.97)	1925 (3.34)	–10.92	0.026
	O10–O16	Oedema, proteinuria and hypertensive disorders in pregnancy, childbirth, and the puerperium	238 (0.33)	247 (0.43)	3.78	0.748
	O20–O29	Other maternal disorders predominantly related to pregnancy	51 (0.07)	45 (0.08)	–11.76	0.565
	O30–O48	Maternal care related to the fetus and amniotic cavity, and possible delivery problems	1841 (2.53)	1701 (2.95)	–7.6	0.123
	O60–O75	Complications of labour and delivery	3118 (4.28)	2987 (5.19)	–4.2	0.331
	O80–O84	Delivery	71 (0.1)	15 (0.03)	–78.87	0.3
	O95–O99	Other obstetric conditions, not elsewhere classified	31 (0.04)	27 (0.05)	–12.9	0.684
	Total		7511 (10.31)	6947 (12.06)	–7.51	0.014
Others symptoms and diseases	R00–R09	Circulatory and respiratory systems	98 (0.13)	77 (0.13)	–21.43	0.253
	R10–R19	Digestive system and abdomen	35 (0.05)	18 (0.03)	–48.57	0.024
	R30–R39	Urinary system	202 (0.28)	162 (0.28)	–19.8	0.025
	R50–R69	General symptoms and signs	144 (0.2)	112 (0.19)	–22.22	0.049
	Total		479 (0.66)	369 (0.64)	–22.96	0.001
Injuries	S00–S09	Injuries to the head	794 (1.09)	493 (0.86)	–37.91	<0.001
	S10–S19	Injuries to the neck	46 (0.06)	22 (0.04)	–52.17	0.005
	S20–S29	Injuries to the thorax	185 (0.25)	111 (0.19)	–40	0.019
	S30–S39	Injuries to the abdomen, lower back, lumbar spine, and pelvis	684 (0.94)	366 (0.64)	–46.49	<0.001
	S40–S49	Injuries to the shoulder and upper arm	2080 (2.86)	1411 (2.45)	–32.16	<0.001
	S50–S59	Injuries to the elbow and forearm	4264 (5.85)	3016 (5.24)	–29.27	<0.001
	S60–S69	Injuries to the wrist and hand	5049 (6.93)	4329 (7.52)	–14.26	0.002
	S70–S79	Injuries to the hip and thigh	11695 (16.06)	9269 (16.1)	–20.74	<0.001
	S80–S89	Injuries to the knee and lower leg	5506 (7.56)	3096 (5.38)	–43.77	<0.001
	S90–S99	Injuries to the ankle and foot	353 (0.48)	277 (0.48)	–21.53	0.005
	T79	Certain early complications of trauma	27 (0.04)	19 (0.03)	–29.63	0.133
	T80–T88	Complications of surgical and medical care, not elsewhere classified	551 (0.76)	368 (0.64)	–33.21	<0.001
	Total		31 234 (42.88)	22777 (39.55)	–27.08	<0.001
Other reasons for admission	Z80–Z99	Persons with potential health hazards related to family and personal history, and certain conditions influencing health status	222 (0.3)	241 (0.42)	8.56	0.592
	Total		222 (0.3)	241 (0.42)	8.56	0.762

Values in parentheses are percentages.

Diseases were classified in 78 blocks of categories. Among these, admissions decreased in 71 categories (91 per cent) and increased in seven (9.0 per cent), although these increases were not significant compared with 2019. Among the most common categories requiring emergency surgery, the greatest reduction was observed for injuries to the knee and lower leg (–43.8 per cent; *P* < 0.001) and injuries to the shoulder and upper arm (–32.2 per cent; *P* < 0.001). An important reduction for diseases of appendix was also observed (–21.0 per cent; *P* < 0.001), and admissions related to disorders of gallbladder, biliary tract, and pancreas decreased by 5.6 per cent, although this was not significantly different from 2019 (*P* = 0.089). Urolithiasis had a moderate increase (0.7 per cent), but the rate was not significantly different from that in 2019 (*P* = 0.860).

Subcategories occurring in at least 400 admissions are reported in *Table 3*, and the complete list is available in *Table S1*. The number of operations for fractures, notably fracture of head and neck of femur (–20.5 per cent), pertrochanteric fracture (–16.8 per cent), fracture of lower leg, including ankle (irrespective of location: –56.0 per cent for upper end of tibia, –53.0 per cent for shaft of tibia, –41.4 per cent for lateral malleolus, –38.5 per cent for other fractures of lower leg) as well as fracture of shoulder and upper arm (upper end of humerus –28.7 per cent, shaft of humerus–36.5 per cent) all decreased significantly compared with 2019.

**Table 3:** Surgical emergencies classified by subcategory (selection of most common)

Chapter	Subcategory code	Subcategory label	Control group (2019)	Lockdown group (2020)	Difference (%)	P*
Circulatory system	I442	Atrioventricular block, complete	750 (1.03)	589 (1.02)	–21.47	0.002
	I743	Embolism and thrombosis of arteries of the lower extremities	450 (0.62)	459 (0.8)	2	0.826
Digestive system	K352	Acute appendicitis with generalized peritonitis	607 (0.83)	531 (0.92)	–12.52	0.184
	K353	Acute appendicitis with localized peritonitis	1945 (2.67)	1588 (2.76)	–18.35	0.002
	K358	Other and unspecified acute appendicitis	2843 (3.9)	2160 (3.75)	–24.02	0.003
	K565	Intestinal adhesions (bands) with obstruction (after infection)	884 (1.21)	721 (1.25)	–18.44	0.008
	K566	Other and unspecified intestinal obstruction	460 (0.63)	394 (0.68)	–14.35	0.152
	K610	Anal abscess	905 (1.24)	679 (1.18)	–24.97	0.004
	K800	Calculus of gallbladder with acute cholecystitis	1507 (2.07)	1411 (2.45)	–6.37	0.67
	K801	Calculus of gallbladder with other cholecystitis	497 (0.68)	433 (0.75)	–12.88	0.159
	K810	Acute cholecystitis	557 (0.76)	526 (0.91)	–5.57	0.231
Skin and subcutaneous tissue	L022	Cutaneous abscess, furuncle, and carbuncle of trunk	456 (0.63)	317 (0.55)	–30.48	0.013
	L024	Cutaneous abscess, furuncle, and carbuncle of limb	589 (0.81)	381 (0.66)	–35.31	<0.001
	L050	Pilonidal cyst and sinus with abscess	744 (1.02)	583 (1.01)	–21.64	0.003
Musculoskeletal system and connective tissue	M650	Abscess of tendon sheath	434 (0.6)	319 (0.55)	–26.5	0.008
	M966	Fracture of bone following insertion of orthopaedic implant, joint prosthesis, or bone plate	558 (0.77)	454 (0.79)	–18.64	0.078
Genitourinary system	N132	Hydronephrosis with renal and ureteral calculous obstruction	584 (0.8)	589 (1.02)	0.86	0.9
	N136	Pyonephrosis	442 (0.61)	414 (0.72)	–6.33	0.326
	N201	Calculus of ureter	1698 (2.33)	1806 (3.14)	6.36	0.393
Pregnancy, childbirth, and the puerperium	O001	Tubal pregnancy	602 (0.83)	516 (0.9)	–14.29	0.09
	O342	Maternal care owing to uterine scar from previous surgery	765 (1.05)	764 (1.33)	–0.13	0.887
	O630	Prolonged first stage (of labour)	410 (0.56)	378 (0.66)	–7.8	0.716
	O680	Labour and delivery complicated by fetal heart rate anomaly	1179 (1.62)	1173 (2.04)	–0.51	0.879
Injuries	S422	Fracture of upper end of humerus	1095 (1.5)	781 (1.36)	–28.68	<0.001
	S423	Fracture of shaft of humerus	551 (0.76)	350 (0.61)	–36.48	0.009
	S520	Fracture of upper end of ulna	431 (0.59)	275 (0.48)	–36.19	<0.001
	S525	Fracture of lower end of radius	2720 (3.73)	2061 (3.58)	–24.23	<0.001
	S526	Fracture of lower end of ulna	496 (0.68)	335 (0.58)	–32.46	0.002
	S626	Fracture of other and unspecified finger(s)	506 (0.69)	331 (0.57)	–34.58	0.002
	S644	Injury of digital nerve of other and unspecified finger	484 (0.66)	472 (0.82)	–2.48	0.64
	S663	Injury of extensor muscle, fascia, and tendon of other and unspecified finger at wrist and hand level	980 (1.35)	917 (1.59)	–6.43	0.096
	S720	Fracture of head and neck of femur	6020 (8.26)	4785 (8.31)	–20.51	<0.001
	S721	Pertrochanteric fracture	3685 (5.06)	3066 (5.32)	–16.8	0.029
	S722	Subtrochanteric fracture of femur	527 (0.72)	403 (0.7)	–23.53	0.118
	S723	Fracture of shaft of femur	681 (0.93)	454 (0.79)	–33.33	<0.001
	S821	Fracture of upper end of tibia	704 (0.97)	310 (0.54)	–55.97	0.001
	S822	Fracture of shaft of tibia	745 (1.02)	350 (0.61)	–53.02	<0.001
	S823	Fracture of lower end of tibia	450 (0.62)	286 (0.5)	–36.44	<0.001
	S826	Fracture of lateral malleolus	541 (0.74)	319 (0.55)	–41.04	<0.001
	S828	Other fractures of lower leg	1750 (2.4)	1084 (1.88)	–38.06	0.001

Values in parentheses are percentages.

### Mortality

Some 2433 deaths (1.87 per cent) were identified in the original population and 2129 (1.87 per cent) in the matched population (*Table S2*). After matching, the overall mortality rate was 1.92 per cent (1096 of 56 982) during the lockdown period and 1.81 per cent (1033 of 56 982) in 2019. The adjusted OR for death in the matched population was 1.06 (95 per cent c.i. 0.97 to 1.15). A significant increase in mortality rate was seen in high-prevalence zones (OR 1.22, 1.06 to 1.40); there were no changes in the low- and intermediate-prevalence zones (*Table 4*).

**Table 4:** Mortality by zone of prevalence of COVID-19 infection

Prevalence zone	Deaths*			Odds ratio^†^
	Control period	**Lockdown period**		
High	374 (1.66)	417 (2.02)		1.22 (1.06, 1.40)
Intermediate	283 (2.01)	303 (2.05)		1.02 (0.87, 1.20)
Low	376 (1.85)	376 (1.75)		0.94 (0.81, 1.09)
Total	1033 (1.81)	1096 (1.92)		1.06 (0.97, 1.15)

Values in parentheses are

*percentages and

^†^95 per cent confidence intervals.

#### Patients with COVID-19

In the subgroup of 863 patients with a diagnosis of COVID-19 infection, the overall mortality rate was 4.0 per cent among those with asymptomatic infection (OR 1.21, 95 per cent c.i. 0.44 to 2.80) and 12.3 per cent for those with symptomatic infection (OR 4.00, 2.60 to 6.32).

## Discussion

This study reports a major decrease in emergency procedures during the COVID-19 pandemic lockdown period in France. The comprehensive data have permitted an in-depth analysis at a national level. There was a 20.9 per cent reduction in emergency surgical admissions to hospital between the 2020 lockdown and the corresponding interval in 2019. Over the weeks after the end of lockdown, no significant difference was observed between the two periods, suggesting a progressive return to usual surgical practices. The decrease in hospital admissions was associated with the regional prevalence of COVID-19, with the greatest reduction seen in the zones of highest prevalence. As no difference was observed between low- and intermediate-COVID-19 prevalence regions, two levels of impact on emergency surgeries were evident: a major impact in high-prevalence regions and a significantly lower level for all other regions. After matching on all available data, in-hospital mortality was slightly and significantly greater in the lockdown group than in the control group in high-prevalence zones. Additionally, the curve for the number of urgent operations week by week during the lockdown was a mirror image of the curve for number of hospital admissions for COVID-19^8^, suggesting that the availability of hospital beds and operating rooms, requisitioned at the peak of the epidemic, had an impact on the operating capacities of the hospitals.

These findings seem to confirm other experiences reported in the media in the early lockdown periods regarding the dramatic and unexpected reduction in non-COVID emergencies^9^^,^^10^.

The present data are consistent with preliminary reports on acute-care surgery in other countries. In Spain, a 60 per cent decrease in acute surgery activity during the acute phase of the pandemic was reported by three tertiary hospitals in Madrid and Barcelona^11^. Similarly, an important reduction in traumatic injuries (almost 38 per cent compared with 2019) was observed in a major trauma centre in the UK^2^. A multicentre study^12^ from 18 general surgery units in a red zone of COVID-19 contagion reported a 45 per cent decrease in admissions for emergency surgical disease and a 41 per cent decrease in operations, despite no discernible differences in overall management approaches to patients who were admitted during the lockdown.

Several factors have been put forward to explain the reduction in emergency surgery. The most common is the patients’ fear of being taken to hospitals receiving people with COVID-19 and the risk of contracting the virus in that environment. This fear has probably been nourished by worrying information transmitted by the media about the situation in hospitals, such as being overwhelmed by patients with COVID and facing equipment shortages including personal protection, and the lack of reassuring messages from hospitals on the management of patients without COVID. Precise reasons for hospital avoidance remain unclear; only indirect evidence is available. A study^13^ from the UK reported that people with low-risk conditions were less likely to present to an emergency department whereas the numbers of non-deferrable emergencies remained constant.

There is already some evidence that avoidance of hospital attendance has led to delayed visits to an emergency department, resulting in more advanced disease. The study^11^ from Spain reported an increased delay of almost 24 h from the onset of symptoms to arrival at a hospital compared with that of a historical control group. A report^3^ from three medical centres in the state of New York found an increase in paediatric perforated appendicitis compared with uncomplicated appendicitis during the surge of COVID-19 outbreak. Similarly, a number of reports have documented decreases in emergency visits for kidney stone disease, with an increase in severe presentations necessitating admission^14^^,^^15^. These data are consistent with the findings of the present study, where there was a moderate increase (0.7 per cent) in the category urolithiasis (N20–N23).

Lockdown restrictions led to unprecedented modifications in lifestyle, resulting in a reduction in road traffic collisions and consequent trauma. In the UK, road casulaties dicreased of 67 per cent compared with 2019^16^. Associations between acute diseases and other lifestyle changes such as food and alcohol consumption, or physical activity, is less straightforward. During the 8-week lockdown in France, a survey of 3000 adults found that men gained an average of 2.7 kg and women 2.3 kg^17^. If short-term weight gain influences the risk of cholecystitis, this might provide partly explain why the reduction in acute cholecystitis (K810, decrease of 5.6 per cent) was relatively modest.

Another issue may have been a shift, when possible, from surgical to medical treatment. This has been suggested for uncomplicated appendicitis or cholecystitis^18^^,^^19^. This might also explain why some disorders for which there is no non-surgical alternative, such as incarcerated hernia or bowel perforation, showed a more moderate reduction^13^. In the absence of evidence of catching up at the end of the lockdown period in the present study, it can be argued that conservative treatment represented a feasible solution for some patients. This warrants further study in relevant conditions.

In many healthcare settings, elective surgery has been severely curtailed. Although this inevitably resulted in fewer complications requiring urgent surgical revision^11^^,^^20^, this must be set against patients listed for elective surgery whose problems deteriorated, leading to an urgent surgical admission. Despite this, the reduction for some conditions remains difficult to explain, in particular for life-threatening diseases such as bowel perforation or incarcerated hernia.

The decrease in admissions for emergencies requiring surgical treatment in the present study was also related to the local prevalence of COVID-19. The analysis highlighted that the decrease in surgical emergencies was identical in zones with a low and intermediate prevalence of COVID-19 infection, and different from that in high-prevalence zones. The mortality rate was also associated with the regional prevalence of hospital admission for COVID-19, with an increased odds of a fatal event. This might suggest that, when a threshold is exceeded in emergency departments, the quality of care may be affected and the mortality rate increases. Previous studies^2^^,^^11^^,^^12^ with contradictory findings may have suffered from having relatively small sample sizes.

The present study has limitations. It was based on an administrative database using classification of disease (ICD-10) codes, rather than on clinical data. Although ICD codes can be extremely accurate, they are not always consistent with clinical classification; for instance, there is no correlation between the Hinchey classification for perforated diverticulitis and ICD codes^21^. The use of a standardized classification does, however, facilitate reproducibility and comparison. Furthermore, admissions were classified only with respect to the main diagnosis, which seemed appropriate for most patients, but could be a simplification for complex emergencies, such as patients with multiple traumatic injuries. No information on conservative treatment in primary or secondary care or medical treatment for surgical emergencies is available. As a result, the decrease in surgical admissions might have overestimated the real incidence of acute surgical conditions. These limitations, however, must be seen in the context of a comprehensive data set at national level which, as a result of using ICD-10 codes, permits comparison with other countries.

The pandemic coupled with a national lockdown had a massive impact on emergency operations, especially in zones with a higher prevalence of COVID-19 infection, where in-hospital mortality increased significantly. Although the surgical community has the ability to adapt and cope with emerging viral infections, such as the human immunodeficiency virus and severe acute respiratory syndrome^21^, it is essential that health authorities act to preserve an adequate workforce, prevent scarcity of resources, and continue to deliver appropriate messages to the public in order to maintain adequate surgical services.

*Disclosure*. All authors delcare no conflict of interest concerning the present study.

## Supplementary material

Supplementary material is available at *BJS Open* online.

## Supplementary Material

zrab039_Supplementary_Data

## Notes

Presented to the Congress of the French Society of Digestive Surgery webinar, November 2020
