# Supplementary material for: Impact of COVID-19 on surgical emergencies: nationwide analysis
Source: BJS Open. 2021 May 22;5(3):zrab039. doi: 10.1093/bjsopen/zrab039 (PMC8140197; doi:10.1093/bjsopen/zrab039)
Supplement: zrab039_Supplementary_Data [file zrab039_supplementary_data.zip › BJS Open-0031 Supporting information Figure S1.docx]

### Supplementary figure S1. Patient Attrition.

##
