# Supplementary material for: Impact of COVID-19 on surgical emergencies: nationwide analysis
Source: BJS Open. 2021 May 22;5(3):zrab039. doi: 10.1093/bjsopen/zrab039 (PMC8140197; doi:10.1093/bjsopen/zrab039)
Supplement: zrab039_Supplementary_Data [file zrab039_supplementary_data.zip › BJS Open-0031 Supporting information Table S1.docx]

### Supplementary table S1. Surgical emergencies classified by subcategory (complete list)

| **Chapter** | **Code** | **Label** | **Control period** | **Lockdown period** | **Change rate (%)** | **P-value** |
| --- | --- | --- | --- | --- | --- | --- |
| Infectious diseases | A410 | Sepsis due to Staphylococcus aureus | 34 (0.05) | 17 (0.03) | -50 | 0.047 |
| Infectious diseases | A415 | Sepsis due to other Gram-negative organisms | 23 (0.03) | 24 (0.04) | 4.35 | 0.873 |
| Infectious diseases | A46 | Erysipelas | 54 (0.07) | 31 (0.05) | -42.59 | 0.048 |
| Neoplasms | C180 | Malignant neoplasm of cecum | 85 (0.12) | 78 (0.14) | -8.24 | 0.812 |
| Neoplasms | C181 | Malignant neoplasm of appendix | 39 (0.05) | 20 (0.03) | -48.72 | 0.034 |
| Neoplasms | C182 | Malignant neoplasm of ascending colon | 103 (0.14) | 88 (0.15) | -14.56 | 0.176 |
| Neoplasms | C183 | Malignant neoplasm of hepatic flexure | 52 (0.07) | 35 (0.06) | -32.69 | 0.074 |
| Neoplasms | C184 | Malignant neoplasm of transverse colon | 51 (0.07) | 55 (0.1) | 7.84 | 0.573 |
| Neoplasms | C185 | Malignant neoplasm of splenic flexure | 38 (0.05) | 44 (0.08) | 15.79 | 0.48 |
| Neoplasms | C186 | Malignant neoplasm of descending colon | 51 (0.07) | 45 (0.08) | -11.76 | 0.608 |
| Neoplasms | C187 | Malignant neoplasm of sigmoid colon | 128 (0.18) | 134 (0.23) | 4.69 | 0.389 |
| Neoplasms | C189 | Malignant neoplasm of colon. unspecified | 33 (0.05) | 31 (0.05) | -6.06 | 0.959 |
| Neoplasms | C19 | Malignant neoplasm of rectosigmoid junction | 40 (0.05) | 45 (0.08) | 12.5 | 0.491 |
| Neoplasms | C20 | Malignant neoplasm of rectum | 51 (0.07) | 66 (0.11) | 29.41 | 0.15 |
| Neoplasms | C56 | Malignant neoplasm of ovary | 65 (0.09) | 42 (0.07) | -35.38 | 0.057 |
| Neoplasms | C61 | Malignant neoplasm of prostate | 38 (0.05) | 42 (0.07) | 10.53 | 0.922 |
| Neoplasms | C64 | Malignant neoplasm of kidney. except renal pelvis | 24 (0.03) | 31 (0.05) | 29.17 | 0.849 |
| Neoplasms | C672 | Malignant neoplasm of lateral wall of bladder | 45 (0.06) | 39 (0.07) | -13.33 | 0.445 |
| Neoplasms | C678 | Malignant neoplasm of overlapping sites of bladder | 36 (0.05) | 55 (0.1) | 52.78 | 0.212 |
| Neoplasms | C679 | Malignant neoplasm of bladder. unspecified | 171 (0.23) | 138 (0.24) | -19.3 | 0.082 |
| Neoplasms | C712 | Malignant neoplasm of temporal lobe | 24 (0.03) | 17 (0.03) | -29.17 | 0.244 |
| Neoplasms | C719 | Malignant neoplasm of brain. unspecified | 23 (0.03) | 17 (0.03) | -26.09 | 0.331 |
| Neoplasms | C786 | Secondary malignant neoplasm of retroperitoneum and peritoneum | 86 (0.12) | 57 (0.1) | -33.72 | 0.004 |
| Neoplasms | C793 | Secondary malignant neoplasm of brain and cerebral meninges | 58 (0.08) | 65 (0.11) | 12.07 | 0.833 |
| Neoplasms | C795 | Secondary malignant neoplasm of bone and bone marrow | 73 (0.1) | 64 (0.11) | -12.33 | 0.524 |
| Neoplasms | C833 | Diffuse large B-cell lymphoma | 47 (0.06) | 42 (0.07) | -10.64 | 0.749 |
| Neoplasms | D27 | Benign neoplasm of ovary | 47 (0.06) | 54 (0.09) | 14.89 | 0.634 |
| Neoplasms | D291 | Benign neoplasm of prostate | 52 (0.07) | 31 (0.05) | -40.38 | 0.292 |
| Nervous system | G060 | Intracranial abscess and granuloma | 25 (0.03) | 18 (0.03) | -28 | 0.481 |
| Nervous system | G551 | Nerve root and plexus compressions in intervertebral disc disorders | 53 (0.07) | 44 (0.08) | -16.98 | 0.176 |
| Nervous system | G560 | Carpal tunnel syndrome | 23 (0.03) | 5 (0.01) | -78.26 | 0.008 |
| Nervous system | G952 | Other and unspecified cord compression | 25 (0.03) | 9 (0.02) | -64 | 0.103 |
| Eye and adnexa | H160 | Corneal ulcer | 28 (0.04) | 20 (0.03) | -28.57 | 0.201 |
| Eye and adnexa | H163 | Interstitial and deep keratitis | 70 (0.1) | 29 (0.05) | -58.57 | 0.005 |
| Eye and adnexa | H192 | Keratitis and keratoconjunctivitis in other infectious and parasitic diseases classified elsewhere | 36 (0.05) | 8 (0.01) | -77.78 | 1 |
| Eye and adnexa | H251 | Age-related nuclear cataract | 26 (0.04) | NA (NA) | NA | 0.014 |
| Eye and adnexa | H330 | Retinal detachment with retinal break | 165 (0.23) | 147 (0.26) | -10.91 | 0.721 |
| Eye and adnexa | H440 | Purulent endophthalmitis | 37 (0.05) | 21 (0.04) | -43.24 | 0.057 |
| Eye and adnexa | H441 | Other endophthalmitis | 33 (0.05) | 10 (0.02) | -69.7 | 0.033 |
| Circulatory system | I200 | Unstable angina | 27 (0.04) | 16 (0.03) | -40.74 | 0.136 |
| Circulatory system | I210 | ST elevation (STEMI) myocardial infarction of anterior wall | 37 (0.05) | 35 (0.06) | -5.41 | 0.755 |
| Circulatory system | I211 | ST elevation (STEMI) myocardial infarction of inferior wall | 37 (0.05) | 33 (0.06) | -10.81 | 0.319 |
| Circulatory system | I214 | Non-ST elevation (NSTEMI) myocardial infarction | 100 (0.14) | 59 (0.1) | -41 | 0.035 |
| Circulatory system | I251 | Atherosclerotic heart disease of native coronary artery | 42 (0.06) | 21 (0.04) | -50 | 0.066 |
| Circulatory system | I255 | Ischemic cardiomyopathy | 42 (0.06) | 23 (0.04) | -45.24 | 0.061 |
| Circulatory system | I313 | Pericardial effusion (noninflammatory) | 42 (0.06) | 37 (0.06) | -11.9 | 0.43 |
| Circulatory system | I330 | Acute and subacute infective endocarditis | 50 (0.07) | 48 (0.08) | -4 | 1 |
| Circulatory system | I350 | Nonrheumatic aortic (valve) stenosis | 28 (0.04) | 13 (0.02) | -53.57 | 0.063 |
| Circulatory system | I440 | Atrioventricular block. first degree | 29 (0.04) | 23 (0.04) | -20.69 | 0.588 |
| Circulatory system | I441 | Atrioventricular block. second degree | 171 (0.23) | 127 (0.22) | -25.73 | 0.096 |
| Circulatory system | I442 | Atrioventricular block. complete | 750 (1.03) | 589 (1.02) | -21.47 | 0.002 |
| Circulatory system | I455 | Other specified heart block | 42 (0.06) | 38 (0.07) | -9.52 | 0.813 |
| Circulatory system | I460 | Cardiac arrest with successful resuscitation | 38 (0.05) | 22 (0.04) | -42.11 | 0.067 |
| Circulatory system | I472 | Ventricular tachycardia | 66 (0.09) | 43 (0.07) | -34.85 | 0.019 |
| Circulatory system | I480 | Paroxysmal atrial fibrillation | 52 (0.07) | 44 (0.08) | -15.38 | 0.5 |
| Circulatory system | I481 | Persistent atrial fibrillation | 27 (0.04) | 11 (0.02) | -59.26 | 0.017 |
| Circulatory system | I482 | Chronic atrial fibrillation | 38 (0.05) | 33 (0.06) | -13.16 | 0.265 |
| Circulatory system | I489 | Unspecified atrial fibrillation and atrial flutter | 29 (0.04) | 14 (0.02) | -51.72 | 0.049 |
| Circulatory system | I495 | Sick sinus syndrome | 234 (0.32) | 180 (0.31) | -23.08 | 0.091 |
| Circulatory system | I500 | Congestive heart failure | 142 (0.19) | 153 (0.27) | 7.75 | 0.753 |
| Circulatory system | I501 | Left ventricular failure. unspecified | 103 (0.14) | 105 (0.18) | 1.94 | 0.972 |
| Circulatory system | I602 | Nontraumatic subarachnoid hemorrhage from anterior communicating artery | 33 (0.05) | 25 (0.04) | -24.24 | 0.444 |
| Circulatory system | I608 | Other nontraumatic subarachnoid hemorrhage | 24 (0.03) | 17 (0.03) | -29.17 | 0.582 |
| Circulatory system | I610 | Nontraumatic intracerebral hemorrhage in hemisphere. subcortical | 34 (0.05) | 28 (0.05) | -17.65 | 0.522 |
| Circulatory system | I620 | Nontraumatic subdural hemorrhage | 96 (0.13) | 72 (0.13) | -25 | 0.219 |
| Circulatory system | I632 | Cerebral infarction due to unspecified occlusion or stenosis of precerebral arteries | 26 (0.04) | 24 (0.04) | -7.69 | 0.963 |
| Circulatory system | I652 | Occlusion and stenosis of carotid artery | 109 (0.15) | 98 (0.17) | -10.09 | 0.57 |
| Circulatory system | I702 | Atherosclerosis of native arteries of the extremities | 301 (0.41) | 252 (0.44) | -16.28 | 0.314 |
| Circulatory system | I710 | Dissection of aorta | 80 (0.11) | 58 (0.1) | -27.5 | 0.13 |
| Circulatory system | I713 | Abdominal aortic aneurysm. ruptured | 42 (0.06) | 27 (0.05) | -35.71 | 0.047 |
| Circulatory system | I724 | Aneurysm of artery of lower extremity | 35 (0.05) | 28 (0.05) | -20 | 0.449 |
| Circulatory system | I742 | Embolism and thrombosis of arteries of the upper extremities | 55 (0.08) | 38 (0.07) | -30.91 | 0.076 |
| Circulatory system | I743 | Embolism and thrombosis of arteries of the lower extremities | 450 (0.62) | 459 (0.8) | 2 | 0.826 |
| Circulatory system | I745 | Embolism and thrombosis of iliac artery | 48 (0.07) | 35 (0.06) | -27.08 | 0.113 |
| Respiratory system | J36 | Peritonsillar abscess | 273 (0.37) | 135 (0.23) | -50.55 | 0.002 |
| Respiratory system | J390 | Retropharyngeal and parapharyngeal abscess | 31 (0.04) | 16 (0.03) | -48.39 | 0.05 |
| Respiratory system | J869 | Pyothorax without fistula | 24 (0.03) | 17 (0.03) | -29.17 | 0.365 |
| Respiratory system | J90 | Pleural effusion. not elsewhere classified | 24 (0.03) | 29 (0.05) | 20.83 | 0.433 |
| Respiratory system | J930 | Spontaneous tension pneumothorax | 32 (0.04) | 20 (0.03) | -37.5 | 0.078 |
| Respiratory system | J931 | Other spontaneous pneumothorax | 100 (0.14) | 62 (0.11) | -38 | 0.023 |
| Respiratory system | J939 | Pneumothorax. unspecified | 29 (0.04) | 25 (0.04) | -13.79 | 0.565 |
| Digestive system | K047 | Periapical abscess without sinus | 74 (0.1) | 67 (0.12) | -9.46 | 0.279 |
| Digestive system | K122 | Cellulitis and abscess of mouth | 204 (0.28) | 98 (0.17) | -51.96 | 0.006 |
| Digestive system | K251 | Acute gastric ulcer with perforation | 60 (0.08) | 64 (0.11) | 6.67 | 0.646 |
| Digestive system | K261 | Acute duodenal ulcer with perforation | 92 (0.13) | 71 (0.12) | -22.83 | 0.058 |
| Digestive system | K265 | Chronic or unspecified duodenal ulcer with perforation | 27 (0.04) | 22 (0.04) | -18.52 | 0.209 |
| Digestive system | K352 | Acute appendicitis with generalized peritonitis | 607 (0.83) | 531 (0.92) | -12.52 | 0.184 |
| Digestive system | K353 | Acute appendicitis with localized peritonitis | 1945 (2.67) | 1588 (2.76) | -18.35 | 0.002 |
| Digestive system | K358 | Other and unspecified acute appendicitis | 2843 (3.9) | 2160 (3.75) | -24.02 | 0.003 |
| Digestive system | K36 | Other appendicitis | 43 (0.06) | 34 (0.06) | -20.93 | 0.329 |
| Digestive system | K37 | Unspecified appendicitis | 82 (0.11) | 44 (0.08) | -46.34 | 0.258 |
| Digestive system | K400 | Bilateral inguinal hernia. with obstruction. without gangrene | 35 (0.05) | 39 (0.07) | 11.43 | 0.663 |
| Digestive system | K403 | Unilateral inguinal hernia. with obstruction. without gangrene | 343 (0.47) | 297 (0.52) | -13.41 | 0.105 |
| Digestive system | K404 | Unilateral inguinal hernia. with gangrene | 39 (0.05) | 20 (0.03) | -48.72 | 0.087 |
| Digestive system | K409 | Unilateral inguinal hernia. without obstruction or gangrene | 170 (0.23) | 146 (0.25) | -14.12 | 0.468 |
| Digestive system | K413 | Unilateral femoral hernia. with obstruction. without gangrene | 133 (0.18) | 95 (0.16) | -28.57 | 0.074 |
| Digestive system | K414 | Unilateral femoral hernia. with gangrene | 30 (0.04) | 17 (0.03) | -43.33 | 0.192 |
| Digestive system | K419 | Unilateral femoral hernia. without obstruction or gangrene | 26 (0.04) | 14 (0.02) | -46.15 | 0.126 |
| Digestive system | K420 | Umbilical hernia with obstruction. without gangrene | 251 (0.34) | 174 (0.3) | -30.68 | 0.056 |
| Digestive system | K421 | Umbilical hernia with gangrene | 23 (0.03) | 19 (0.03) | -17.39 | 0.326 |
| Digestive system | K429 | Umbilical hernia without obstruction or gangrene | 68 (0.09) | 40 (0.07) | -41.18 | 0.458 |
| Digestive system | K430 | Incisional hernia with obstruction. without gangrene | 229 (0.31) | 159 (0.28) | -30.57 | 0.01 |
| Digestive system | K431 | Incisional hernia with gangrene | 31 (0.04) | 19 (0.03) | -38.71 | 0.136 |
| Digestive system | K432 | Incisional hernia without obstruction or gangrene | 65 (0.09) | 51 (0.09) | -21.54 | 0.179 |
| Digestive system | K436 | Other and unspecified ventral hernia with obstruction. without gangrene | 59 (0.08) | 42 (0.07) | -28.81 | 0.141 |
| Digestive system | K439 | Ventral hernia without obstruction or gangrene | 39 (0.05) | 28 (0.05) | -28.21 | 0.679 |
| Digestive system | K440 | Diaphragmatic hernia with obstruction. without gangrene | 42 (0.06) | 27 (0.05) | -35.71 | 0.14 |
| Digestive system | K460 | Unspecified abdominal hernia with obstruction. without gangrene | 31 (0.04) | 22 (0.04) | -29.03 | 0.094 |
| Digestive system | K550 | Acute vascular disorders of intestine | 194 (0.27) | 151 (0.26) | -22.16 | 0.11 |
| Digestive system | K561 | Intussusception | 32 (0.04) | 23 (0.04) | -28.12 | 0.178 |
| Digestive system | K562 | Volvulus | 233 (0.32) | 193 (0.34) | -17.17 | 0.07 |
| Digestive system | K563 | Gallstone ileus | 25 (0.03) | 14 (0.02) | -44 | 0.105 |
| Digestive system | K564 | Other impaction of intestine | 38 (0.05) | 34 (0.06) | -10.53 | 0.691 |
| Digestive system | K565 | Intestinal adhesions [bands] with obstruction (postinfection) | 884 (1.21) | 721 (1.25) | -18.44 | 0.008 |
| Digestive system | K566 | Other and unspecified intestinal obstruction | 460 (0.63) | 394 (0.68) | -14.35 | 0.152 |
| Digestive system | K570 | Diverticulitis of small intestine with perforation and abscess | 35 (0.05) | 19 (0.03) | -45.71 | 0.024 |
| Digestive system | K572 | Diverticulitis of large intestine with perforation and abscess | 291 (0.4) | 252 (0.44) | -13.4 | 0.575 |
| Digestive system | K573 | Diverticular disease of large intestine without perforation or abscess | 23 (0.03) | 23 (0.04) | 0 | 0.928 |
| Digestive system | K574 | Diverticulitis of both small and large intestine with perforation and abscess | 26 (0.04) | 15 (0.03) | -42.31 | 0.134 |
| Digestive system | K603 | Anal fistula | 90 (0.12) | 54 (0.09) | -40 | 0.037 |
| Digestive system | K610 | Anal abscess | 905 (1.24) | 679 (1.18) | -24.97 | 0.004 |
| Digestive system | K611 | Rectal abscess | 23 (0.03) | 21 (0.04) | -8.7 | 0.647 |
| Digestive system | K612 | Anorectal abscess | 44 (0.06) | 23 (0.04) | -47.73 | 0.04 |
| Digestive system | K613 | Ischiorectal abscess | 33 (0.05) | 23 (0.04) | -30.3 | 0.464 |
| Digestive system | K625 | Hemorrhage of anus and rectum | 37 (0.05) | 23 (0.04) | -37.84 | 0.069 |
| Digestive system | K631 | Perforation of intestine (nontraumatic) | 89 (0.12) | 85 (0.15) | -4.49 | 0.813 |
| Digestive system | K632 | Fistula of intestine | 40 (0.05) | 22 (0.04) | -45 | 0.122 |
| Digestive system | K643 | Fourth degree hemorrhoids | 26 (0.04) | 20 (0.03) | -23.08 | 0.53 |
| Digestive system | K650 | Generalized (acute) peritonitis | 389 (0.53) | 281 (0.49) | -27.76 | 0.02 |
| Digestive system | K658 | Other peritonitis | 58 (0.08) | 21 (0.04) | -63.79 | 0.006 |
| Digestive system | K661 | Hemoperitoneum | 47 (0.06) | 41 (0.07) | -12.77 | 0.315 |
| Digestive system | K800 | Calculus of gallbladder with acute cholecystitis | 1507 (2.07) | 1411 (2.45) | -6.37 | 0.67 |
| Digestive system | K801 | Calculus of gallbladder with other cholecystitis | 497 (0.68) | 433 (0.75) | -12.88 | 0.159 |
| Digestive system | K802 | Calculus of gallbladder without cholecystitis | 125 (0.17) | 137 (0.24) | 9.6 | 0.131 |
| Digestive system | K803 | Calculus of bile duct with cholangitis | 75 (0.1) | 80 (0.14) | 6.67 | 0.844 |
| Digestive system | K804 | Calculus of bile duct with cholecystitis | 154 (0.21) | 137 (0.24) | -11.04 | 0.504 |
| Digestive system | K805 | Calculus of bile duct without cholangitis or cholecystitis | 68 (0.09) | 57 (0.1) | -16.18 | 0.503 |
| Digestive system | K810 | Acute cholecystitis | 557 (0.76) | 526 (0.91) | -5.57 | 0.231 |
| Digestive system | K811 | Chronic cholecystitis | 81 (0.11) | 79 (0.14) | -2.47 | 0.752 |
| Digestive system | K821 | Hydrops of gallbladder | 30 (0.04) | 41 (0.07) | 36.67 | 0.36 |
| Digestive system | K851 | Biliary acute pancreatitis | 198 (0.27) | 206 (0.36) | 4.04 | 0.896 |
| Digestive system | K913 | Postprocedural intestinal obstruction | 41 (0.06) | 27 (0.05) | -34.15 | 0.143 |
| Digestive system | K914 | Colostomy and enterostomy malfunction | 34 (0.05) | 19 (0.03) | -44.12 | 0.019 |
| Skin and subcutaneous tissue | L021 | Cutaneous abscess. furuncle and carbuncle of neck | 28 (0.04) | 35 (0.06) | 25 | 0.454 |
| Skin and subcutaneous tissue | L022 | Cutaneous abscess. furuncle and carbuncle of trunk | 456 (0.63) | 317 (0.55) | -30.48 | 0.013 |
| Skin and subcutaneous tissue | L023 | Cutaneous abscess. furuncle and carbuncle of buttock | 258 (0.35) | 124 (0.22) | -51.94 | 0.003 |
| Skin and subcutaneous tissue | L024 | Cutaneous abscess. furuncle and carbuncle of limb | 589 (0.81) | 381 (0.66) | -35.31 | <0.001 |
| Skin and subcutaneous tissue | L028 | Cutaneous abscess. furuncle and carbuncle of other sites | 52 (0.07) | 18 (0.03) | -65.38 | 0.005 |
| Skin and subcutaneous tissue | L029 | Cutaneous abscess. furuncle and carbuncle. unspecified | 44 (0.06) | 20 (0.03) | -54.55 | 0.231 |
| Skin and subcutaneous tissue | L030 | Cellulitis and acute lymphangitis of finger and toe | 144 (0.2) | 83 (0.14) | -42.36 | 0.002 |
| Skin and subcutaneous tissue | L031 | Cellulitis and acute lymphangitis of other parts of limb | 55 (0.08) | 46 (0.08) | -16.36 | 0.184 |
| Skin and subcutaneous tissue | L050 | Pilonidal cyst and sinus with abscess | 744 (1.02) | 583 (1.01) | -21.64 | 0.003 |
| Skin and subcutaneous tissue | L059 | Pilonidal cyst and sinus without abscess | 110 (0.15) | 111 (0.19) | 0.91 | 0.972 |
| Skin and subcutaneous tissue | L088 | Other specified local infections of the skin and subcutaneous tissue | 77 (0.11) | 55 (0.1) | -28.57 | 0.036 |
| Skin and subcutaneous tissue | L720 | Epidermal cyst | 33 (0.05) | 26 (0.05) | -21.21 | 0.433 |
| Skin and subcutaneous tissue | L721 | Pilar and trichodermal cyst | 54 (0.07) | 38 (0.07) | -29.63 | 0.037 |
| Skin and subcutaneous tissue | L732 | Hidradenitis suppurativa | 50 (0.07) | 40 (0.07) | -20 | 0.261 |
| Skin and subcutaneous tissue | L893 | Pressure ulcer of buttock | 36 (0.05) | 22 (0.04) | -38.89 | 0.055 |
| Skin and subcutaneous tissue | L97 | Non-pressure chronic ulcer of lower limb. not elsewhere classified | 87 (0.12) | 74 (0.13) | -14.94 | 0.476 |
| Musculoskeletal system and connective tissue | M000 | Staphylococcal arthritis and polyarthritis | 334 (0.46) | 226 (0.39) | -32.34 | 0.003 |
| Musculoskeletal system and connective tissue | M002 | Other streptococcal arthritis and polyarthritis | 79 (0.11) | 77 (0.13) | -2.53 | 0.458 |
| Musculoskeletal system and connective tissue | M008 | Arthritis and polyarthritis due to other bacteria | 101 (0.14) | 89 (0.15) | -11.88 | 0.446 |
| Musculoskeletal system and connective tissue | M009 | Pyogenic arthritis. unspecified | 124 (0.17) | 91 (0.16) | -26.61 | 0.034 |
| Musculoskeletal system and connective tissue | M161 | Unilateral primary osteoarthritis of hip | 37 (0.05) | 12 (0.02) | -67.57 | 0.048 |
| Musculoskeletal system and connective tissue | M232 | Derangement of meniscus due to old tear or injury | 29 (0.04) | 6 (0.01) | -79.31 | 0.008 |
| Musculoskeletal system and connective tissue | M480 | Spinal stenosis | 46 (0.06) | 47 (0.08) | 2.17 | 0.905 |
| Musculoskeletal system and connective tissue | M511 | Thoracic. thoracolumbar and lumbosacral intervertebral disc disorders with radiculopathy | 156 (0.21) | 173 (0.3) | 10.9 | 0.431 |
| Musculoskeletal system and connective tissue | M512 | Other thoracic. thoracolumbar and lumbosacral intervertebral disc displacement | 68 (0.09) | 53 (0.09) | -22.06 | 0.44 |
| Musculoskeletal system and connective tissue | M650 | Abscess of tendon sheath | 434 (0.6) | 319 (0.55) | -26.5 | 0.008 |
| Musculoskeletal system and connective tissue | M651 | Other infective (teno)synovitis | 88 (0.12) | 72 (0.13) | -18.18 | 0.284 |
| Musculoskeletal system and connective tissue | M658 | Other synovitis and tenosynovitis | 78 (0.11) | 84 (0.15) | 7.69 | 0.75 |
| Musculoskeletal system and connective tissue | M705 | Other bursitis of knee | 26 (0.04) | 15 (0.03) | -42.31 | 0.09 |
| Musculoskeletal system and connective tissue | M711 | Other infective bursitis | 23 (0.03) | 23 (0.04) | 0 | 0.972 |
| Musculoskeletal system and connective tissue | M726 | Necrotizing fasciitis | 53 (0.07) | 55 (0.1) | 3.77 | 0.75 |
| Musculoskeletal system and connective tissue | M795 | Residual foreign body in soft tissue | 269 (0.37) | 255 (0.44) | -5.2 | 0.734 |
| Musculoskeletal system and connective tissue | M808 | Other osteoporosis with current pathological fracture | 25 (0.03) | 13 (0.02) | -48 | 0.076 |
| Musculoskeletal system and connective tissue | M841 | Nonunion of fracture [pseudarthrosis] | 35 (0.05) | 19 (0.03) | -45.71 | 0.028 |
| Musculoskeletal system and connective tissue | M844 | Pathological fracture. not elsewhere classified | 43 (0.06) | 44 (0.08) | 2.33 | 1 |
| Musculoskeletal system and connective tissue | M853 | Osteitis condensans | 23 (0.03) | 6 (0.01) | -73.91 | 0.015 |
| Musculoskeletal system and connective tissue | M861 | Other acute osteomyelitis | 126 (0.17) | 62 (0.11) | -50.79 | 0.002 |
| Musculoskeletal system and connective tissue | M864 | Chronic osteomyelitis with draining sinus | 23 (0.03) | 15 (0.03) | -34.78 | 0.415 |
| Musculoskeletal system and connective tissue | M866 | Other chronic osteomyelitis | 29 (0.04) | 21 (0.04) | -27.59 | 0.138 |
| Musculoskeletal system and connective tissue | M868 | Other osteomyelitis | 43 (0.06) | 39 (0.07) | -9.3 | 0.959 |
| Musculoskeletal system and connective tissue | M869 | Osteomyelitis. unspecified | 122 (0.17) | 74 (0.13) | -39.34 | 0.037 |
| Musculoskeletal system and connective tissue | M907 | Fracture of bone in neoplastic disease | 94 (0.13) | 71 (0.12) | -24.47 | 0.325 |
| Musculoskeletal system and connective tissue | M966 | Fracture of bone following insertion of orthopedic implant. joint prosthesis. or bone plate | 558 (0.77) | 454 (0.79) | -18.64 | 0.078 |
| Genitourinary system | N029 | Recurrent and persistent hematuria with unspecified morphologic changes | 24 (0.03) | 14 (0.02) | -41.67 | 0.179 |
| Genitourinary system | N10 | Acute pyelonephritis | 276 (0.38) | 229 (0.4) | -17.03 | 0.22 |
| Genitourinary system | N111 | Chronic obstructive pyelonephritis | 218 (0.3) | 205 (0.36) | -5.96 | 0.816 |
| Genitourinary system | N130 | Hydronephrosis with ureteropelvic junction obstruction | 87 (0.12) | 105 (0.18) | 20.69 | 0.285 |
| Genitourinary system | N131 | Hydronephrosis with ureteral stricture. not elsewhere classified | 111 (0.15) | 96 (0.17) | -13.51 | 0.343 |
| Genitourinary system | N132 | Hydronephrosis with renal and ureteral calculous obstruction | 584 (0.8) | 589 (1.02) | 0.86 | 0.9 |
| Genitourinary system | N133 | Other and unspecified hydronephrosis | 83 (0.11) | 76 (0.13) | -8.43 | 0.615 |
| Genitourinary system | N136 | Pyonephrosis | 442 (0.61) | 414 (0.72) | -6.33 | 0.326 |
| Genitourinary system | N178 | Other acute kidney failure | 75 (0.1) | 79 (0.14) | 5.33 | 0.392 |
| Genitourinary system | N179 | Acute kidney failure. unspecified | 29 (0.04) | 37 (0.06) | 27.59 | 0.44 |
| Genitourinary system | N185 | Chronic kidney disease. stage 5 | 55 (0.08) | 23 (0.04) | -58.18 | 0.026 |
| Genitourinary system | N200 | Calculus of kidney | 244 (0.33) | 229 (0.4) | -6.15 | 0.299 |
| Genitourinary system | N201 | Calculus of ureter | 1698 (2.33) | 1806 (3.14) | 6.36 | 0.393 |
| Genitourinary system | N202 | Calculus of kidney with calculus of ureter | 231 (0.32) | 191 (0.33) | -17.32 | 0.124 |
| Genitourinary system | N210 | Calculus in bladder | 28 (0.04) | 14 (0.02) | -50 | 0.131 |
| Genitourinary system | N23 | Unspecified renal colic | 371 (0.51) | 350 (0.61) | -5.66 | 0.722 |
| Genitourinary system | N288 | Other specified disorders of kidney and ureter | 27 (0.04) | 32 (0.06) | 18.52 | 0.719 |
| Genitourinary system | N320 | Bladder-neck obstruction | 25 (0.03) | 12 (0.02) | -52 | 0.047 |
| Genitourinary system | N328 | Other specified disorders of bladder | 38 (0.05) | 31 (0.05) | -18.42 | 0.443 |
| Genitourinary system | N390 | Urinary tract infection. site not specified | 27 (0.04) | 13 (0.02) | -51.85 | 0.04 |
| Genitourinary system | N40 | Benign prostatic hyperplasia | 84 (0.12) | 48 (0.08) | -42.86 | 0.061 |
| Genitourinary system | N44 | Noninflammatory disorders of testis | 281 (0.39) | 235 (0.41) | -16.37 | 0.051 |
| Genitourinary system | N450 | Orchitis. epididymitis and epididymo-orchitis with abscess | 33 (0.05) | 23 (0.04) | -30.3 | 0.319 |
| Genitourinary system | N459 | Inflammatory disorders of male genital organs. not elsewhere classified | 32 (0.04) | 18 (0.03) | -43.75 | 0.03 |
| Genitourinary system | N47 | Disorders of prepuce | 30 (0.04) | 12 (0.02) | -60 | 0.066 |
| Genitourinary system | N492 | Inflammatory disorders of scrotum | 114 (0.16) | 67 (0.12) | -41.23 | 0.003 |
| Genitourinary system | N498 | Inflammatory disorders of other specified male genital organs | 28 (0.04) | 18 (0.03) | -35.71 | 0.209 |
| Genitourinary system | N508 | Other specified disorders of male genital organs | 40 (0.05) | 25 (0.04) | -37.5 | 0.215 |
| Genitourinary system | N61 | Inflammatory disorders of breast | 24 (0.03) | 21 (0.04) | -12.5 | 0.828 |
| Genitourinary system | N700 | Acute salpingitis and oophoritis | 167 (0.23) | 157 (0.27) | -5.99 | 0.489 |
| Genitourinary system | N733 | Female acute pelvic peritonitis | 46 (0.06) | 30 (0.05) | -34.78 | 0.108 |
| Genitourinary system | N751 | Abscess of Bartholin's gland | 315 (0.43) | 208 (0.36) | -33.97 | 0.002 |
| Genitourinary system | N758 | Other diseases of Bartholin's gland | 98 (0.13) | 72 (0.13) | -26.53 | 0.086 |
| Genitourinary system | N764 | Abscess of vulva | 94 (0.13) | 72 (0.13) | -23.4 | 0.037 |
| Genitourinary system | N830 | Follicular cyst of ovary | 30 (0.04) | 21 (0.04) | -30 | 0.326 |
| Genitourinary system | N831 | Corpus luteum cyst | 37 (0.05) | 27 (0.05) | -27.03 | 0.217 |
| Genitourinary system | N832 | Other and unspecified ovarian cysts | 144 (0.2) | 96 (0.17) | -33.33 | 0.025 |
| Genitourinary system | N835 | Torsion of ovary. ovarian pedicle and fallopian tube | 157 (0.22) | 129 (0.22) | -17.83 | 0.092 |
| Genitourinary system | N938 | Other specified abnormal uterine and vaginal bleeding | 40 (0.05) | 20 (0.03) | -50 | 0.019 |
| Pregnancy. childbirth and the puerperium | O001 | Tubal pregnancy | 602 (0.83) | 516 (0.9) | -14.29 | 0.09 |
| Pregnancy. childbirth and the puerperium | O008 | Other ectopic pregnancy | 86 (0.12) | 91 (0.16) | 5.81 | 0.336 |
| Pregnancy. childbirth and the puerperium | O009 | Ectopic pregnancy. unspecified | 194 (0.27) | 174 (0.3) | -10.31 | 0.459 |
| Pregnancy. childbirth and the puerperium | O020 | Blighted ovum and nonhydatidiform mole | 38 (0.05) | 17 (0.03) | -55.26 | 0.24 |
| Pregnancy. childbirth and the puerperium | O021 | Missed abortion | 139 (0.19) | 126 (0.22) | -9.35 | 0.775 |
| Pregnancy. childbirth and the puerperium | O031 | Delayed or excessive hemorrhage following incomplete spontaneous abortion | 268 (0.37) | 260 (0.45) | -2.99 | 0.775 |
| Pregnancy. childbirth and the puerperium | O033 | Other and unspecified complications following incomplete spontaneous abortion | 35 (0.05) | 26 (0.05) | -25.71 | 0.782 |
| Pregnancy. childbirth and the puerperium | O034 | Incomplete spontaneous abortion without complication | 355 (0.49) | 272 (0.47) | -23.38 | 0.393 |
| Pregnancy. childbirth and the puerperium | O036 | Delayed or excessive hemorrhage following complete or unspecified spontaneous abortion | 67 (0.09) | 62 (0.11) | -7.46 | 0.849 |
| Pregnancy. childbirth and the puerperium | O039 | Complete or unspecified spontaneous abortion without complication | 150 (0.21) | 163 (0.28) | 8.67 | 0.23 |
| Pregnancy. childbirth and the puerperium | O041 | Incomplete. complicated by delayed or excessive haemorrhage medical abortion | 31 (0.04) | 38 (0.07) | 22.58 | 0.239 |
| Pregnancy. childbirth and the puerperium | O044 | Incomplete. without complication medical abortion | 53 (0.07) | 41 (0.07) | -22.64 | 0.278 |
| Pregnancy. childbirth and the puerperium | O049 | Complete or unspecified. without complication medical abortion | 65 (0.09) | 79 (0.14) | 21.54 | 0.414 |
| Pregnancy. childbirth and the puerperium | O071 | Delayed or excessive hemorrhage following failed attempted termination of pregnancy | 23 (0.03) | 12 (0.02) | -47.83 | 0.06 |
| Pregnancy. childbirth and the puerperium | O081 | Delayed or excessive hemorrhage following ectopic and molar pregnancy | 55 (0.08) | 48 (0.08) | -12.73 | 0.591 |
| Pregnancy. childbirth and the puerperium | O140 | Mild to moderate pre-eclampsia | 79 (0.11) | 81 (0.14) | 2.53 | 0.959 |
| Pregnancy. childbirth and the puerperium | O141 | Severe pre-eclampsia | 119 (0.16) | 125 (0.22) | 5.04 | 0.753 |
| Pregnancy. childbirth and the puerperium | O142 | HELLP syndrome | 40 (0.05) | 41 (0.07) | 2.5 | 0.408 |
| Pregnancy. childbirth and the puerperium | O244 | Gestational diabetes mellitus | 51 (0.07) | 45 (0.08) | -11.76 | 0.347 |
| Pregnancy. childbirth and the puerperium | O300 | Twin pregnancy | 60 (0.08) | 53 (0.09) | -11.67 | 0.284 |
| Pregnancy. childbirth and the puerperium | O321 | Maternal care for breech presentation | 256 (0.35) | 249 (0.43) | -2.73 | 0.691 |
| Pregnancy. childbirth and the puerperium | O322 | Maternal care for transverse and oblique lie | 35 (0.05) | 23 (0.04) | -34.29 | 0.053 |
| Pregnancy. childbirth and the puerperium | O331 | Maternal care for disproportion due to generally contracted pelvis | 23 (0.03) | 21 (0.04) | -8.7 | 0.713 |
| Pregnancy. childbirth and the puerperium | O342 | Maternal care due to uterine scar from previous surgery | 765 (1.05) | 764 (1.33) | -0.13 | 0.887 |
| Pregnancy. childbirth and the puerperium | O363 | Maternal care for signs of fetal hypoxia | 158 (0.22) | 121 (0.21) | -23.42 | 0.054 |
| Pregnancy. childbirth and the puerperium | O365 | Maternal care for known or suspected poor fetal growth | 87 (0.12) | 66 (0.11) | -24.14 | 0.311 |
| Pregnancy. childbirth and the puerperium | O366 | Maternal care for excessive fetal growth | 36 (0.05) | 30 (0.05) | -16.67 | 0.398 |
| Pregnancy. childbirth and the puerperium | O368 | Maternal care for other specified fetal problems | 31 (0.04) | 20 (0.03) | -35.48 | 0.136 |
| Pregnancy. childbirth and the puerperium | O411 | Infection of amniotic sac and membranes | 40 (0.05) | 21 (0.04) | -47.5 | 0.026 |
| Pregnancy. childbirth and the puerperium | O420 | Premature rupture of membranes. onset of labor within 24 hours of rupture | 64 (0.09) | 56 (0.1) | -12.5 | 0.504 |
| Pregnancy. childbirth and the puerperium | O421 | Premature rupture of membranes. onset of labor more than 24 hours following rupture | 88 (0.12) | 93 (0.16) | 5.68 | 0.565 |
| Pregnancy. childbirth and the puerperium | O440 | Complete placenta previa NOS or without hemorrhage | 26 (0.04) | 32 (0.06) | 23.08 | 0.295 |
| Pregnancy. childbirth and the puerperium | O441 | Complete placenta previa with hemorrhage | 79 (0.11) | 74 (0.13) | -6.33 | 0.874 |
| Pregnancy. childbirth and the puerperium | O458 | Other premature separation of placenta | 31 (0.04) | 31 (0.05) | 0 | 0.856 |
| Pregnancy. childbirth and the puerperium | O48 | Late pregnancy | 62 (0.09) | 47 (0.08) | -24.19 | 0.312 |
| Pregnancy. childbirth and the puerperium | O601 | Preterm labor with preterm delivery | 69 (0.09) | 64 (0.11) | -7.25 | 0.689 |
| Pregnancy. childbirth and the puerperium | O603 | Preterm delivery without spontaneous labour | 47 (0.06) | 55 (0.1) | 17.02 | 0.436 |
| Pregnancy. childbirth and the puerperium | O610 | Failed medical induction of labor | 200 (0.27) | 186 (0.32) | -7 | 0.303 |
| Pregnancy. childbirth and the puerperium | O620 | Primary inadequate contractions | 58 (0.08) | 55 (0.1) | -5.17 | 0.918 |
| Pregnancy. childbirth and the puerperium | O621 | Secondary uterine inertia | 199 (0.27) | 178 (0.31) | -10.55 | 0.222 |
| Pregnancy. childbirth and the puerperium | O628 | Other abnormalities of forces of labor | 29 (0.04) | 10 (0.02) | -65.52 | 0.042 |
| Pregnancy. childbirth and the puerperium | O630 | Prolonged first stage (of labor) | 410 (0.56) | 378 (0.66) | -7.8 | 0.716 |
| Pregnancy. childbirth and the puerperium | O631 | Prolonged second stage (of labor) | 97 (0.13) | 105 (0.18) | 8.25 | 0.587 |
| Pregnancy. childbirth and the puerperium | O640 | Obstructed labor due to incomplete rotation of fetal head | 42 (0.06) | 36 (0.06) | -14.29 | 0.693 |
| Pregnancy. childbirth and the puerperium | O641 | Obstructed labor due to breech presentation | 123 (0.17) | 126 (0.22) | 2.44 | 0.474 |
| Pregnancy. childbirth and the puerperium | O648 | Obstructed labor due to other malposition and malpresentation | 28 (0.04) | 19 (0.03) | -32.14 | 0.266 |
| Pregnancy. childbirth and the puerperium | O654 | Obstructed labor due to fetopelvic disproportion. unspecified | 47 (0.06) | 45 (0.08) | -4.26 | 0.659 |
| Pregnancy. childbirth and the puerperium | O655 | Obstructed labor due to abnormality of maternal pelvic organs | 56 (0.08) | 57 (0.1) | 1.79 | 1 |
| Pregnancy. childbirth and the puerperium | O664 | Failed trial of labor | 40 (0.05) | 22 (0.04) | -45 | 0.095 |
| Pregnancy. childbirth and the puerperium | O665 | Attempted application of vacuum extractor and forceps | 31 (0.04) | 29 (0.05) | -6.45 | 0.837 |
| Pregnancy. childbirth and the puerperium | O668 | Other specified obstructed labor | 54 (0.07) | 51 (0.09) | -5.56 | 0.796 |
| Pregnancy. childbirth and the puerperium | O680 | Labour and delivery complicated by fetal heart rate anomaly | 1179 (1.62) | 1173 (2.04) | -0.51 | 0.879 |
| Pregnancy. childbirth and the puerperium | O681 | Labour and delivery complicated by meconium in amniotic fluid | 35 (0.05) | 29 (0.05) | -17.14 | 0.404 |
| Pregnancy. childbirth and the puerperium | O682 | Labour and delivery complicated by fetal heart rate anomaly with meconium in amniotic fluid | 311 (0.43) | 312 (0.54) | 0.32 | 0.587 |
| Pregnancy. childbirth and the puerperium | O690 | Labor and delivery complicated by prolapse of cord | 36 (0.05) | 38 (0.07) | 5.56 | 0.797 |
| Pregnancy. childbirth and the puerperium | O722 | Delayed and secondary postpartum hemorrhage | 27 (0.04) | 19 (0.03) | -29.63 | 0.081 |
| Pregnancy. childbirth and the puerperium | O800 | Spontaneous vertex delivery | 71 (0.1) | 15 (0.03) | -78.87 | 0.474 |
| Pregnancy. childbirth and the puerperium | O998 | Other specified diseases and conditions complicating pregnancy. childbirth and the puerperium | 31 (0.04) | 27 (0.05) | -12.9 | 0.633 |
| Others symptoms and diseases | R02 | Gangrene. not elsewhere classified | 75 (0.1) | 64 (0.11) | -14.67 | 0.395 |
| Others symptoms and diseases | R040 | Epistaxis | 23 (0.03) | 13 (0.02) | -43.48 | 0.504 |
| Others symptoms and diseases | R104 | Other and unspecified abdominal pain | 35 (0.05) | 18 (0.03) | -48.57 | 0.079 |
| Others symptoms and diseases | R31 | Hematuria | 165 (0.23) | 125 (0.22) | -24.24 | 0.012 |
| Others symptoms and diseases | R33 | Retention of urine | 37 (0.05) | 37 (0.06) | 0 | 0.887 |
| Others symptoms and diseases | R55 | Syncope and collapse | 68 (0.09) | 60 (0.1) | -11.76 | 0.528 |
| Others symptoms and diseases | R570 | Cardiogenic shock | 38 (0.05) | 26 (0.05) | -31.58 | 0.122 |
| Others symptoms and diseases | R572 | Septic shock | 38 (0.05) | 26 (0.05) | -31.58 | 0.135 |
| Injuries | S011 | Open wound of eyelid and periocular area | 143 (0.2) | 97 (0.17) | -32.17 | 0.014 |
| Injuries | S023 | Fracture of orbital floor | 35 (0.05) | 22 (0.04) | -37.14 | 0.528 |
| Injuries | S024 | Fracture of malar. maxillary and zygoma bones | 33 (0.05) | 17 (0.03) | -48.48 | 0.056 |
| Injuries | S026 | Fracture of mandible | 172 (0.24) | 94 (0.16) | -45.35 | 0.065 |
| Injuries | S055 | Penetrating wound with foreign body of eyeball | 39 (0.05) | 24 (0.04) | -38.46 | 0.194 |
| Injuries | S056 | Penetrating wound without foreign body of eyeball | 76 (0.1) | 51 (0.09) | -32.89 | 0.096 |
| Injuries | S064 | Epidural hemorrhage | 35 (0.05) | 18 (0.03) | -48.57 | 0.078 |
| Injuries | S065 | Traumatic subdural hemorrhage | 217 (0.3) | 147 (0.26) | -32.26 | 0.003 |
| Injuries | S067 | Intracranial injury with prolonged coma | 44 (0.06) | 23 (0.04) | -47.73 | 0.122 |
| Injuries | S122 | Fracture of third cervical vertebra | 46 (0.06) | 22 (0.04) | -52.17 | 0.009 |
| Injuries | S220 | Fracture of thoracic vertebra | 132 (0.18) | 75 (0.13) | -43.18 | 0.058 |
| Injuries | S221 | Multiple fractures of thoracic spine | 28 (0.04) | 21 (0.04) | -25 | 0.5 |
| Injuries | S271 | Traumatic hemothorax | 25 (0.03) | 15 (0.03) | -40 | 0.066 |
| Injuries | S301 | Contusion of abdominal wall | 28 (0.04) | 8 (0.01) | -71.43 | 0.008 |
| Injuries | S302 | Contusion of external genital organs | 44 (0.06) | 20 (0.03) | -54.55 | 0.065 |
| Injuries | S311 | Open wound of abdominal wall without penetration into peritoneal cavity | 24 (0.03) | 16 (0.03) | -33.33 | 0.098 |
| Injuries | S314 | Open wound of vagina and vulva | 41 (0.06) | 22 (0.04) | -46.34 | 0.074 |
| Injuries | S320 | Fracture of lumbar vertebra | 235 (0.32) | 159 (0.28) | -32.34 | 0.041 |
| Injuries | S324 | Fracture of acetabulum | 58 (0.08) | 23 (0.04) | -60.34 | 0.004 |
| Injuries | S327 | Multiple fractures of lumbar spine and pelvis | 41 (0.06) | 31 (0.05) | -24.39 | 0.383 |
| Injuries | S360 | Injury of spleen | 59 (0.08) | 29 (0.05) | -50.85 | 0.037 |
| Injuries | S361 | Injury of liver and gallbladder and bile duct | 32 (0.04) | 12 (0.02) | -62.5 | 0.01 |
| Injuries | S364 | Injury of small intestine | 45 (0.06) | 22 (0.04) | -51.11 | 0.058 |
| Injuries | S365 | Injury of colon | 41 (0.06) | 10 (0.02) | -75.61 | <0.001 |
| Injuries | S368 | Injury of other intra-abdominal organs | 36 (0.05) | 14 (0.02) | -61.11 | 0.141 |
| Injuries | S420 | Fracture of clavicle | 160 (0.22) | 94 (0.16) | -41.25 | 0.017 |
| Injuries | S422 | Fracture of upper end of humerus | 1095 (1.5) | 781 (1.36) | -28.68 | <0.001 |
| Injuries | S423 | Fracture of shaft of humerus | 551 (0.76) | 350 (0.61) | -36.48 | 0.009 |
| Injuries | S424 | Fracture of lower end of humerus | 242 (0.33) | 174 (0.3) | -28.1 | 0.018 |
| Injuries | S431 | Subluxation and dislocation of acromioclavicular joint | 32 (0.04) | 12 (0.02) | -62.5 | 0.009 |
| Injuries | S520 | Fracture of upper end of ulna | 431 (0.59) | 275 (0.48) | -36.19 | <0.001 |
| Injuries | S521 | Fracture of upper end of radius | 133 (0.18) | 98 (0.17) | -26.32 | 0.059 |
| Injuries | S522 | Fracture of shaft of ulna | 79 (0.11) | 43 (0.07) | -45.57 | 0.003 |
| Injuries | S523 | Fracture of shaft of radius | 119 (0.16) | 70 (0.12) | -41.18 | 0.017 |
| Injuries | S524 | Fracture of shafts of both ulna and radius | 150 (0.21) | 75 (0.13) | -50 | <0.001 |
| Injuries | S525 | Fracture of lower end of radius | 2720 (3.73) | 2061 (3.58) | -24.23 | <0.001 |
| Injuries | S526 | Fracture of lower end of ulna | 496 (0.68) | 335 (0.58) | -32.46 | 0.002 |
| Injuries | S527 | Multiple fractures of forearm | 39 (0.05) | 15 (0.03) | -61.54 | 0.015 |
| Injuries | S528 | Fracture of other parts of forearm | 53 (0.07) | 17 (0.03) | -67.92 | 0.012 |
| Injuries | S531 | Subluxation and dislocation of ulnohumeral joint | 44 (0.06) | 27 (0.05) | -38.64 | 0.098 |
| Injuries | S610 | Open wound of thumb without damage to nail | 196 (0.27) | 152 (0.26) | -22.45 | 0.657 |
| Injuries | S611 | Open wound of thumb with damage to nail | 71 (0.1) | 59 (0.1) | -16.9 | 0.29 |
| Injuries | S618 | Open wound of other parts of wrist and hand | 70 (0.1) | 71 (0.12) | 1.43 | 0.888 |
| Injuries | S620 | Fracture of navicular [scaphoid] bone of wrist | 34 (0.05) | 23 (0.04) | -32.35 | 0.282 |
| Injuries | S622 | Fracture of first metacarpal bone | 55 (0.08) | 21 (0.04) | -61.82 | 0.01 |
| Injuries | S623 | Fracture of other and unspecified metacarpal bone | 378 (0.52) | 268 (0.47) | -29.1 | 0.003 |
| Injuries | S624 | Multiple fractures of metacarpal bones | 67 (0.09) | 45 (0.08) | -32.84 | 0.13 |
| Injuries | S625 | Fracture of thumb | 100 (0.14) | 67 (0.12) | -33 | 0.032 |
| Injuries | S626 | Fracture of other and unspecified finger(s) | 506 (0.69) | 331 (0.57) | -34.58 | 0.002 |
| Injuries | S627 | Multiple fractures of fingers | 58 (0.08) | 48 (0.08) | -17.24 | 0.559 |
| Injuries | S628 | Fracture of other and unspecified parts of wrist and hand | 101 (0.14) | 87 (0.15) | -13.86 | 0.441 |
| Injuries | S630 | Subluxation and dislocation of wrist and hand joints | 41 (0.06) | 22 (0.04) | -46.34 | 0.094 |
| Injuries | S631 | Subluxation and dislocation of thumb | 69 (0.09) | 59 (0.1) | -14.49 | 0.457 |
| Injuries | S634 | Traumatic rupture of ligament of finger at metacarpophalangeal and interphalangeal joint(s) | 35 (0.05) | 22 (0.04) | -37.14 | 0.151 |
| Injuries | S636 | Other and unspecified sprain of finger(s) | 25 (0.03) | 9 (0.02) | -64 | 0.09 |
| Injuries | S640 | Injury of ulnar nerve at wrist and hand level | 29 (0.04) | 53 (0.09) | 82.76 | 0.011 |
| Injuries | S641 | Injury of median nerve at wrist and hand level | 43 (0.06) | 27 (0.05) | -37.21 | 0.042 |
| Injuries | S642 | Injury of radial nerve at wrist and hand level | 56 (0.08) | 46 (0.08) | -17.86 | 0.322 |
| Injuries | S643 | Injury of digital nerve of thumb | 106 (0.15) | 75 (0.13) | -29.25 | 0.106 |
| Injuries | S644 | Injury of digital nerve of other and unspecified finger | 484 (0.66) | 472 (0.82) | -2.48 | 0.64 |
| Injuries | S651 | Injury of radial artery at wrist and hand level | 66 (0.09) | 96 (0.17) | 45.45 | 0.439 |
| Injuries | S655 | Injury of blood vessel of other and unspecified finger | 67 (0.09) | 68 (0.12) | 1.49 | 0.539 |
| Injuries | S660 | Injury of long flexor muscle. fascia and tendon of thumb at wrist and hand level | 62 (0.09) | 61 (0.11) | -1.61 | 0.977 |
| Injuries | S661 | Injury of flexor muscle. fascia and tendon of other and unspecified finger at wrist and hand level | 327 (0.45) | 294 (0.51) | -10.09 | 0.499 |
| Injuries | S662 | Injury of extensor muscle. fascia and tendon of thumb at wrist and hand level | 231 (0.32) | 198 (0.34) | -14.29 | 0.124 |
| Injuries | S663 | Injury of extensor muscle. fascia and tendon of other and unspecified finger at wrist and hand level | 980 (1.35) | 917 (1.59) | -6.43 | 0.096 |
| Injuries | S666 | Injury of multiple flexor muscles and tendons at wrist and hand level | 64 (0.09) | 72 (0.13) | 12.5 | 0.395 |
| Injuries | S667 | Injury of multiple extensor muscles and tendons at wrist and hand level | 62 (0.09) | 73 (0.13) | 17.74 | 0.864 |
| Injuries | S668 | Injury of other specified muscles. fascia and tendons at wrist and hand level | 86 (0.12) | 59 (0.1) | -31.4 | 0.283 |
| Injuries | S670 | Crushing injury of thumb | 73 (0.1) | 72 (0.13) | -1.37 | 0.893 |
| Injuries | S680 | Traumatic metacarpophalangeal amputation of thumb | 63 (0.09) | 76 (0.13) | 20.63 | 0.75 |
| Injuries | S681 | Traumatic metacarpophalangeal amputation of other and unspecified finger | 325 (0.45) | 286 (0.5) | -12 | 0.108 |
| Injuries | S682 | Traumatic amputation of two or more fingers alone (complete)(partial) | 63 (0.09) | 53 (0.09) | -15.87 | 0.067 |
| Injuries | S697 | Multiple injuries of wrist and hand | 56 (0.08) | 47 (0.08) | -16.07 | 0.439 |
| Injuries | S701 | Contusion of thigh | 39 (0.05) | 20 (0.03) | -48.72 | 0.049 |
| Injuries | S720 | Fracture of head and neck of femur | 6020 (8.26) | 4785 (8.31) | -20.51 | <0.001 |
| Injuries | S721 | Pertrochanteric fracture | 3685 (5.06) | 3066 (5.32) | -16.8 | 0.029 |
| Injuries | S722 | Subtrochanteric fracture of femur | 527 (0.72) | 403 (0.7) | -23.53 | 0.118 |
| Injuries | S723 | Fracture of shaft of femur | 681 (0.93) | 454 (0.79) | -33.33 | <0.001 |
| Injuries | S724 | Fracture of lower end of femur | 360 (0.49) | 274 (0.48) | -23.89 | 0.003 |
| Injuries | S727 | Multiple fractures of femur | 73 (0.1) | 40 (0.07) | -45.21 | 0.053 |
| Injuries | S728 | Other fracture of femur | 122 (0.17) | 74 (0.13) | -39.34 | 0.009 |
| Injuries | S729 | Unspecified fracture of femur | 48 (0.07) | 29 (0.05) | -39.58 | 0.107 |
| Injuries | S730 | Subluxation and dislocation of hip | 25 (0.03) | 23 (0.04) | -8 | 0.842 |
| Injuries | S761 | Injury of quadriceps muscle. fascia and tendon | 115 (0.16) | 101 (0.18) | -12.17 | 0.548 |
| Injuries | S801 | Contusion of lower leg | 71 (0.1) | 46 (0.08) | -35.21 | 0.027 |
| Injuries | S818 | Open wound of lower leg | 24 (0.03) | 30 (0.05) | 25 | 0.436 |
| Injuries | S820 | Fracture of patella | 303 (0.42) | 197 (0.34) | -34.98 | 0.01 |
| Injuries | S821 | Fracture of upper end of tibia | 704 (0.97) | 310 (0.54) | -55.97 | 0.001 |
| Injuries | S822 | Fracture of shaft of tibia | 745 (1.02) | 350 (0.61) | -53.02 | <0.001 |
| Injuries | S823 | Fracture of lower end of tibia | 450 (0.62) | 286 (0.5) | -36.44 | <0.001 |
| Injuries | S824 | Fracture of shaft of fibula | 54 (0.07) | 29 (0.05) | -46.3 | 0.098 |
| Injuries | S825 | Fracture of medial malleolus | 205 (0.28) | 140 (0.24) | -31.71 | 0.035 |
| Injuries | S826 | Fracture of lateral malleolus | 541 (0.74) | 319 (0.55) | -41.04 | <0.001 |
| Injuries | S827 | Multiple fractures of lower leg | 236 (0.32) | 136 (0.24) | -42.37 | 0.007 |
| Injuries | S828 | Other fractures of lower leg | 1750 (2.4) | 1084 (1.88) | -38.06 | 0.001 |
| Injuries | S829 | Unspecified fracture of lower leg | 39 (0.05) | 12 (0.02) | -69.23 | 0.105 |
| Injuries | S860 | Injury of Achilles tendon | 327 (0.45) | 117 (0.2) | -64.22 | <0.001 |
| Injuries | S862 | Injury of muscle(s) and tendon(s) of anterior muscle group at lower leg level | 57 (0.08) | 40 (0.07) | -29.82 | 0.097 |
| Injuries | S920 | Fracture of calcaneus | 124 (0.17) | 79 (0.14) | -36.29 | 0.023 |
| Injuries | S921 | Fracture of talus | 27 (0.04) | 16 (0.03) | -40.74 | 0.09 |
| Injuries | S923 | Fracture of metatarsal bone(s) | 75 (0.1) | 61 (0.11) | -18.67 | 0.304 |
| Injuries | S924 | Fracture of great toe | 34 (0.05) | 34 (0.06) | 0 | 0.949 |
| Injuries | S927 | Multiple fractures of foot | 28 (0.04) | 16 (0.03) | -42.86 | 0.06 |
| Injuries | S961 | Injury of muscle and tendon of long extensor muscle of toe at ankle and foot level | 65 (0.09) | 71 (0.12) | 9.23 | 0.393 |
| Injuries | T793 | Post-traumatic wound infection. not elsewhere classified | 27 (0.04) | 19 (0.03) | -29.63 | 0.066 |
| Injuries | T810 | Haemorrhage and haematoma complicating a procedure. not elsewhere classified | 76 (0.1) | 38 (0.07) | -50 | 0.025 |
| Injuries | T813 | Disruption of wound. not elsewhere classified | 104 (0.14) | 53 (0.09) | -49.04 | 0.004 |
| Injuries | T821 | Mechanical complication of cardiac electronic device | 23 (0.03) | 21 (0.04) | -8.7 | 1 |
| Injuries | T831 | Mechanical complication of other urinary devices and implants | 25 (0.03) | 26 (0.05) | 4 | 0.874 |
| Injuries | T840 | Mechanical complication of internal joint prosthesis | 249 (0.34) | 190 (0.33) | -23.69 | 0.024 |
| Injuries | T841 | Mechanical complication of internal fixation device of bones of limb | 25 (0.03) | 15 (0.03) | -40 | 0.25 |
| Injuries | T845 | Infection and inflammatory reaction due to internal joint prosthesis | 26 (0.04) | 19 (0.03) | -26.92 | 0.441 |
| Injuries | T855 | Mechanical complication of gastrointestinal prosthetic devices. implants and grafts | 23 (0.03) | 6 (0.01) | -73.91 | 0.062 |
| Other reasons for admission | Z450 | Encounter for adjustment and management of cardiac device | 34 (0.05) | 29 (0.05) | -14.71 | 0.714 |
| Other reasons for admission | Z458 | Encounter for adjustment and management of other implanted devices | 80 (0.11) | 74 (0.13) | -7.5 | 0.894 |
| Other reasons for admission | Z470 | Poisoning by agents primarily affecting the gastrointestinal system. histamine H2-receptor antagonists | 85 (0.12) | 117 (0.2) | 37.65 | 0.9 |
| Other reasons for admission | Z488 | Encounter for other specified postprocedural aftercare | 23 (0.03) | 21 (0.04) | -8.7 | 0.593 |
