# Supplementary material for: Impact of COVID-19 on surgical emergencies: nationwide analysis
Source: BJS Open. 2021 May 22;5(3):zrab039. doi: 10.1093/bjsopen/zrab039 (PMC8140197; doi:10.1093/bjsopen/zrab039)
Supplement: zrab039_Supplementary_Data [file zrab039_supplementary_data.zip › BJS Open-0031 Supporting information Table S2.docx]

### Supplementary table S2. Baseline characteristics for the matched population.

| **Covariates** | **Control group (2019)** | **Lockdown group (2020)** | **SMD** |
| --- | --- | --- | --- |
|  | **n=57583** | **n=57583** |  |
| Sex. female | 32269 (56.6) | 32269 (56.6) | <0.001 |
| Age. mean (SD) | 57.12 (23.14) | 57.35 (23.05) | 0.01 |
| Age |  |  | 0.034 |
| <30 | 9927 (17.4) | 9409 (16.5) |  |
| 30-40 | 8162 (14.3) | 8680 (15.2) |  |
| 40-50 | 5527 ( 9.7) | 5534 ( 9.7) |  |
| 50-60 | 6183 (10.9) | 6176 (10.8) |  |
| 60-75 | 11297 (19.8) | 11072 (19.4) |  |
| >75 | 15886 (27.9) | 16111 (28.3) |  |
| Charlson Score. mean (sd) | 0.43 (0.86) | 0.44 (0.88) | 0.011 |
| Charlson Score |  |  | 0.009 |
| 0 | 42140 (74.0) | 41960 (73.6) |  |
| 1-2 | 12568 (22.1) | 12662 (22.2) |  |
| >3 | 2274 ( 4.0) | 2360 ( 4.1) |  |
| Myocardial infarction | 661 ( 1.2) | 646 ( 1.1) | 0.002 |
| Congestive heart failure | 3002 ( 5.3) | 3071 ( 5.4) | 0.005 |
| Peripheral vascular disease | 1668 ( 2.9) | 1687 ( 3.0) | 0.002 |
| Cerebrovascular disease | 1337 ( 2.3) | 1334 ( 2.3) | <0.001 |
| Dementia | 2458 ( 4.3) | 2387 ( 4.2) | 0.006 |
| Chronic pulmonary disease | 1916 ( 3.4) | 2188 ( 3.8) | 0.026 |
| Rheumatic disease | 262 ( 0.5) | 296 ( 0.5) | 0.009 |
| Peptic ulcer disease | 331 ( 0.6) | 292 ( 0.5) | 0.009 |
| Mild liver disease | 518 ( 0.9) | 590 ( 1.0) | 0.013 |
| Diabetes without chronic complication | 4059 ( 7.1) | 4159 ( 7.3) | 0.007 |
| Diabetes with chronic complication | 1095 ( 1.9) | 983 ( 1.7) | 0.015 |
| Hemiplegia or paraplegia | 973 ( 1.7) | 990 ( 1.7) | 0.002 |
| Renal disease | 1998 ( 3.5) | 2074 ( 3.6) | 0.007 |
| Any malignancy. including lymphoma and leukemia. except malignant neoplasm of skin | 2752 ( 4.8) | 2853 ( 5.0) | 0.008 |
| Moderate or severe liver disease | 120 ( 0.2) | 150 ( 0.3) | 0.011 |
| Metastatic solid tumor | 1062 ( 1.9) | 1079 ( 1.9) | 0.002 |
| AIDS/HIV | 60 ( 0.1) | 51 ( 0.1) | 0.005 |
